# Expansion of *PmBEAT* genes in the *Prunus mume* genome induces characteristic floral scent production

**DOI:** 10.1038/s41438-018-0104-4

**Published:** 2019-02-01

**Authors:** Fei Bao, Anqi Ding, Tengxun Zhang, Le Luo, Jia Wang, Tangren Cheng, Qixiang Zhang

**Affiliations:** 1 Beijing Key Laboratory of Ornamental Plants Germplasm Innovation & Molecular Breeding, National Engineering Research Center for Floriculture, Beijing Laboratory of Urban and Rural Ecological Environment, Engineering Research Center of Landscape Environment of Ministry of Education, Key Laboratory of Genetics and Breeding in Forest Trees and Ornamental Plants of Ministry of Education, School of Landscape Architecture, Beijing Forestry University, Beijing, 100083 China; 2 Beijing Advanced Innovation Center for Tree Breeding by Molecular Design, Beijing Forestry University, Beijing, 100083 China

**Keywords:** Plant evolution, Plant molecular biology

## Abstract

*Prunus mume* is the only plant in the genus *Prunus* of the Rosaceae family with a characteristic floral scent, and the main component of this scent is benzyl acetate. By contrast, benzyl acetate is not synthesized in *Prunus persica* flowers. Here, we searched for *benzyl alcohol acetyltransferase* (*BEAT*) genes based on genomic data from *P. mume* and *P. persica* and found 44 unique *PmBEATs* in *P. mume*. These genes, which were mainly detected in clusters on chromosomes, originated from gene duplication events during the species evolution of *P. mume*, and retroduplication and tandem duplication were the two dominant duplication patterns. The genes *PmBEAT34*, *PmBEAT36* and *PmBEAT37*, which were generated by tandem duplication, were highly expressed in flowers, and their highest levels were detected during the blooming stage. In vitro, PmBEAT34, PmBEAT3, and PmBEAT37 all had benzyl alcohol acetyltransferase activity that was localized in the cytoplasm. Overexpression of the *PmBEAT36* or *PmBEAT37* genes increased benzyl acetate production in the petal protoplasts of *P. mume*, and interference in the expression of these genes slightly decreased the benzyl acetate content. In addition, light and temperature regulated the expression of the *PmBEAT34*, *PmBEAT36* and *PmBEAT37* genes. According to these results, we hypothesize that the expansion of the *PmBEAT* genes in the genome induce the characteristic floral scent of *P. mume*.

## Chinese plum: Unraveling the genetics of scent

*Prunus mume*, Chinese plum, owes its unique scent to extra gene copies. Researchers knew the main component of *P. mume* blooms’ prized scent, but the details of its production were unknown. Qixiang Zhang at the Beijing Forestry University and co-workers investigated the genetic basis of the scent, and uncovered an interesting evolutionary tale. A search for the scent production genes showed that *P. mume* has 44 copies; closely related *P. persica*, which lacks the special scent, has only two. Further analysis showed that the genes are clustered together, indicating they originated from duplication events, the evolutionary equivalent of accidental copying. The superfluous copies, released from their usual responsibilities, were free to evolve new functions, including scent production. These results illuminate how new plant traits can evolve, and unravel the mystery of this special scent.

## Introduction

*Prunus mume* (mei) is a traditional flower in China that was domesticated more than 3000 years ago as an ornamental and fruit plant. Mei belongs to the *Prunus* species of the Rosaceae family, and the flowers open in early spring. Mei flowers have a unique aroma compared to other plants in *Prunus*, such as peach, apricot and plum. *P. mume* originates from the southwestern part of China and the Yangtze River basin. The Yangtze River basin, which is in the subtropical monsoon climate zone, is the main *P. mume* cultivation area, and the flowering time is usually between January and February, when the daily average temperature ranges from 4 to 10 °C. The flowers are believed to have a richer fragrance in the evening and in slightly cold weather. However, no studies have rigorously examined how light or temperature affects the synthesis of flower scent in *P. mume*.

Throughout the long cultivation history of *P. mume* in China, breeders have produced many different varieties. Although these varieties have differences in scent that are detectable by the human nose, benzyl acetate has been reported to be the main component of the fragrance of *P. mume*^1^. Benzyl acetate is synthesized by the phenylpropanoid pathway. The terminal reaction utilizes benzyl alcohol and acetyl coenzyme A (acetyl-CoA) as substrates. During catalysis by acetyl-CoA/benzyl alcohol acetyltransferase (BEAT), the acetyl group of acetyl-CoA is transferred to the carbonyl group of benzyl alcohol to generate benzyl acetate. The first benzyl alcohol acetyltransferase was reported in *Clarkia breweri* (CbBEAT), in which the major floral scent constituent is also benzyl acetate. Some other alcohols have been shown to be catalyzed by CbBEAT; however, the highest catalytic activity occurred when benzyl alcohol was the substrate^2^. Aharoni et al.^3^ identified an *alcohol acyltransferase* gene in strawberry (*SAAT*) and showed that SAAT could utilize multiple alcohols as substrates, including benzyl alcohol. An *alcohol acetyltransferase 1* (*RhAAT1*) gene has also been characterized in roses. Additionally, overexpression of this *RhAAT1* gene in transgenic petunia plants was shown to produce higher levels of benzyl acetate, although RhAAT1 displayed higher acetyltransferase activity when geraniol was the substrate than when aromatic alcohols were the substrate in vitro^4,5^. Several types of alcohol acyltransferases have been reported in different species of plants, and they all can use a broad range of acyl-CoAs and alcohols as substrates. Some examples are anthraniloyl-CoA:methanol acyltransferase (AMAT), which is responsible for the formation of methyl anthranilate in Washington Concord grapes; acetyl-CoA:(Z)−3-hexen-1-ol acetyltransferase (CHAT), which is responsible for the production of (Z)−3-hexen-1-yl acetate in *Arabidopsis*; and coniferyl alcohol acyltransferase (PhCFAT), which is responsible for the synthesis of coniferyl acetate in petunia^6–12^. The motifs H*XXX*D and DFGWG are considered to be highly conserved in CoA-dependent acyltransferases^13^. The H*XXX*D motif is located in the center of the reaction channel of the protein structure, and the DFGWG motif is considered indispensable for catalysis. Another conserved motif, LS*X*TL*XXX*Y*XXX*G, plays an important role in reactions that use acetyl-CoA as a cosubstrate^3^.

Gene duplication is a form of mutation in which a genomic region is replicated. Gene duplication plays an important role in species evolution because it provides raw materials for the evolution of new genes and new genetic functions. Multiple mechanisms contribute to gene duplication, including tandem duplication, segmental duplication, transposon-mediated duplication and retroduplication. Tandem duplication results from unequal crossing-over events and generates tandemly arrayed paralogous genes^14^. Segmental duplication originates from rearranged genomic regions after whole-genome duplications and occurs most frequently in plants^15^. In transposon-mediated duplication, genes are usually captured by Mutator-like elements^16,17^. Retroduplication is another transposable element (TE)-associated mechanism that occurs when the messenger RNA (mRNA) of an expressed gene is reverse transcribed to DNA and then inserted into the genome^18^. These duplicate genes are referred to as retrogenes and usually contain no introns. Because the regulatory sequences in the promoter are usually not duplicated, most retrogenes are believed not to be functional. However, some evidence suggests that retrogenes are functional in plants. Wang et al.^19^ found some retrogenes in rice to be under selection^19^. Retrogene transcripts and coding proteins have been detected in the rice and *Arabidopsis SKP1* gene family^20,21^. In addition, approximately one-fourth and one-third of retrogenes in rice and *Arabidopsis*, respectively, exhibit expression patterns similar to their source genes^22,23^.

We sequenced and assembled the genome of *P. mume* in 2012. Based on genomic data, we showed that the *BEAT* gene family had expanded notably in *P. mume*, compared with *Malus*×*domestics* and *Fragaria vesca* in Rosaceae; additionally, most of the *BEAT* genes were clustered, suggesting that these *BEAT* genes originated from serial duplication events^24^. Here, we have further characterized the *BEAT* genes in *P. mume*. We identified a group of *PmBEAT* genes that was expanded in the *P. mume* genome compared to that of *Prunus persica*, which does not exhibit the benzyl acetate synthetic pathway in flowers. We selected the *PmBEAT* genes with high expression levels in flowers and analyzed their spatiotemporal expression patterns and benzyl alcohol acetyltransferase activities, and the results demonstrate the important role of *PmBEAT* genes in the synthesis of benzoyl acetate. Finally, we explored how light or temperature can affect the benzyl acetate synthesis pathway.

## Results

### Benzyl alcohol acetyltransferase activity in the flowers of *P. mume*

**Fig. 1:**
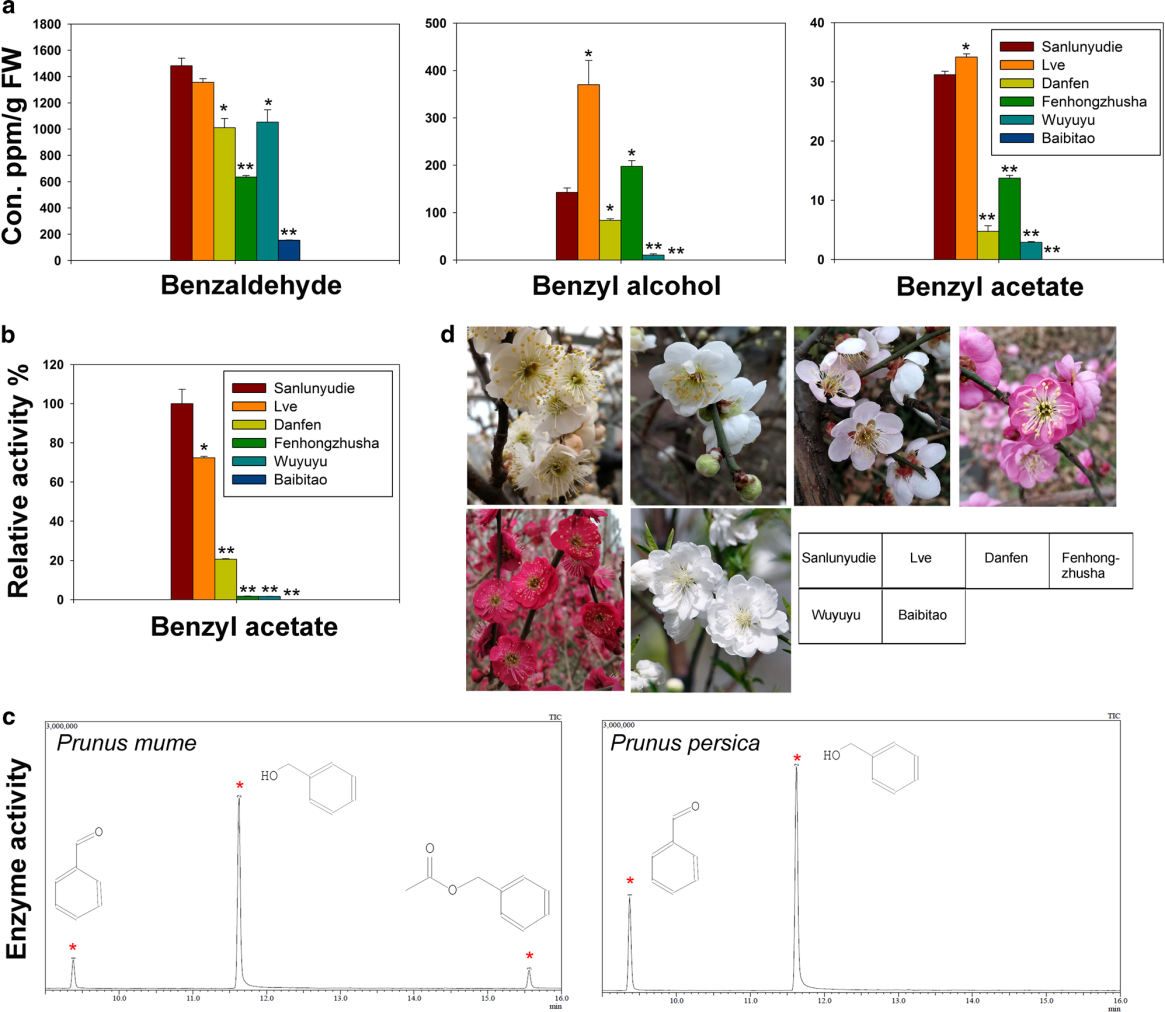
Analyses of endogenous benzyl acetate content and benzyl alcohol acetyltransferase activity in *P. mume* and *P. persica*. **a** Analysis of the contents of endogenous benzaldehyde, benzyl alcohol and benzyl acetate in the blooming flowers of different varieties of *P. mume* and *P. persica* using GC-MS. Benzyl propionate was used as the internal standard for quantitative analysis. The data are presented as the mean values of three replicates ± SD. **b** The relative activities of benzyl alcohol acetyltransferase in the blooming flowers of *P. mume* and *P. persica*. Data are presented as the mean values of three replicates ± SD. *P < 0.05 and **P < 0.01 (Student’s *t*-test). Three independent experiments were performed with similar results. **c** The gas chromatograms of *P. mume* (‘Sanlunyudie’) and *P. persica* (‘Baibitao’) from enzyme activity analysis. Benzyl alcohol was supplied in excess in the experiment. The peaks corresponding to benzaldehyde, benzyl alcohol and benzyl acetate are indicated with red asterisks. **d** Flowers of different varieties of *P. mume* and *P. persica*, including ‘Sanlunyudie’, ‘Lve’, ‘Danfen’, ‘Fenhongzhusha’ and ‘Wuyuyu’ (varieties of *P. mume*), and ‘Baibitao’ (a variety of *P. persica*)

In earlier research, we found that the dominant compound responsible for the fragrance of *P. mume* flowers was ester benzyl acetate^25^, which is synthesized by the benzenoid/phenylpropanoid pathway. CbBEAT in *C. breweri* has been reported to catalyze the synthesis of benzyl acetate using benzyl alcohol and acetyl-CoA as substrates, and the substrate benzyl alcohol has been shown to be synthesized from the reduction of benzaldehyde in plants^2,10^. Differences exist in the content of endogenous benzyl acetate in the flowers of different *P. mume* varieties. The content in the white flower varieties ‘Sanlunyudie’ and ‘Lve’ is significantly higher than that in the red flower varieties ‘Danfen’, ‘Fenhongzhusha’ and ‘Wuyuyu’ (Fig. 1a, d). In addition, the contents of benzaldehyde and benzyl alcohol differ among varieties, but the differences do not correspond to the flower colors. We extracted the total protein of the flower for analysis of the benzyl alcohol acetyltransferase activity in vitro. The results showed that enzyme activity in the white flower varieties was significantly higher than that in the red flower varieties (Fig. 1b). These results suggest that the content of benzyl alcohol and the activity of benzyl alcohol acetyltransferase are two important factors that determine the benzyl acetate content in different varieties. *P. mume* and *P. persica* both belong to the *Prunus* genus of the Rosaceae family. However, no benzyl alcohol or benzyl acetate was detected in the flowers of *P. persica* ‘Baibitao’ (Fig. 1a, d). Additionally, benzyl acetate could not be synthesized in ‘Baibitao’ when benzyl alcohol was supplied for enzyme activity analysis (Fig. 1b, c). These results indicate that the synthesis efficiency differs among the varieties of *P. mume* and that the pathway for the synthesis of benzyl acetate with benzyl alcohol as the substrate does not exist in the flowers of *P. persica*.

### Identification of the *BEAT* genes in the *Prunus mume* genome

**Fig. 2:**
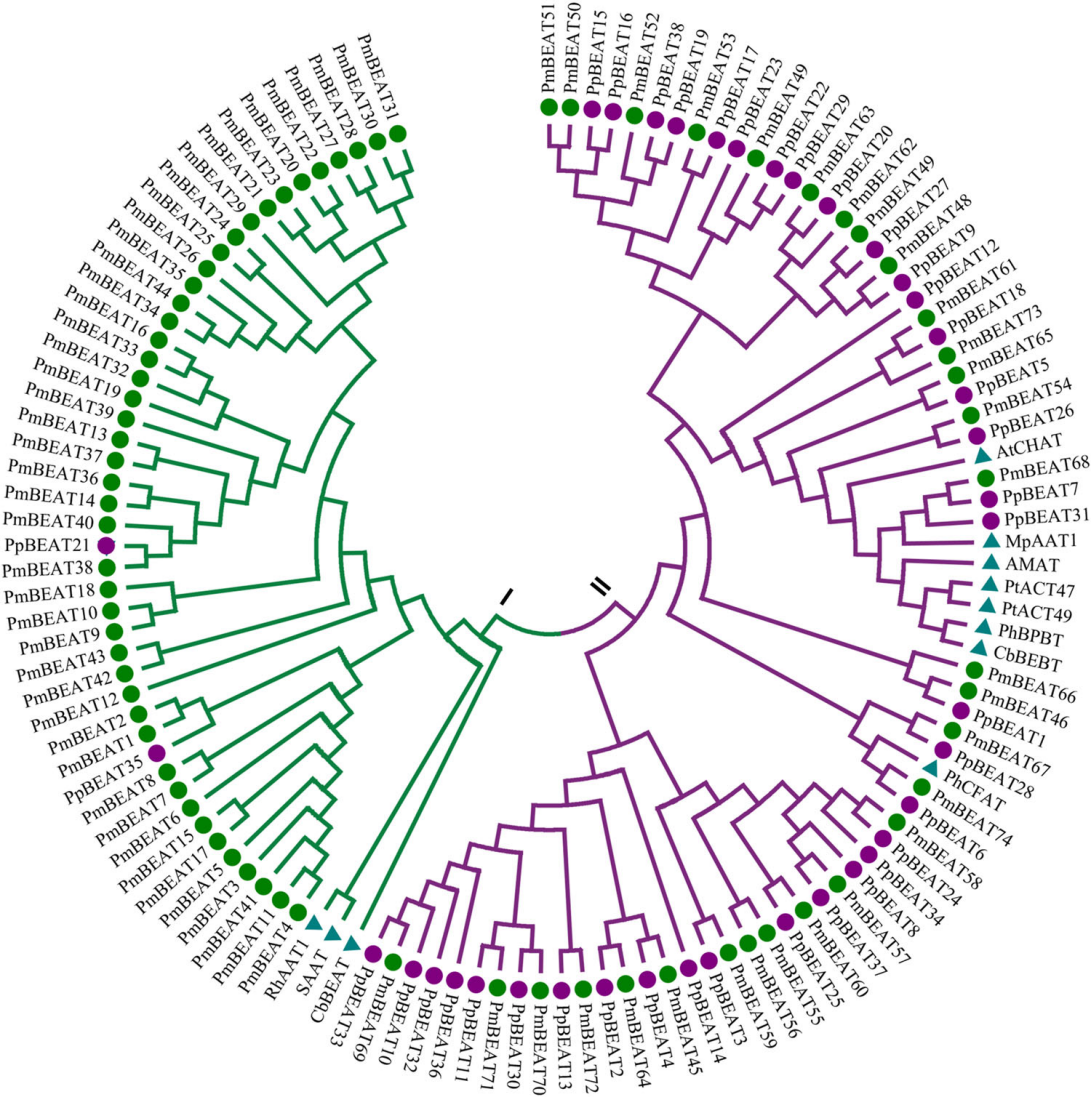
Phylogenetic tree of benzyl alcohol acetyltransferases obtained from *P. mume* and *P. persica* genomes and previously reported alcohol acetyltransferases. The tree was generated using MEGA 6 software with the neighbor-joining method. The green solid points denote benzyl alcohol acetyltransferases from *P. mume*, the purple solid points denote benzyl alcohol acetyltransferases from *P. persica* and the blue triangles denote reported alcohol acetyltransferases from other species

To identify the *BEAT* genes in the genome, BLASTP was performed using the amino acid sequence of CbBEAT as a query. In total, this method yielded 74 genes from the *P. mume* genome and 38 genes from the *P. persica* genome. The evolutionary history was inferred, and a phylogenetic tree was constructed that included other reported alcohol acetyltransferases (Fig. 2). Cluster analysis showed that CbBEAT, SAAT, RhAAT1 and the 44 PmBEATs were all in group I, which also included 2 PpBEATs from *P. persica*. PpBEAT35 has the highest sequence similarity with PmBEAT1 and PmBEAT2, both with 48% identity. PpBEAT21 has the highest sequence similarity with PmBEAT38, with differences in only 24 amino acids (95% identity) (Fig. S1). In group II, 30 PmBEATs and 36 PpBEATs were distributed evenly throughout the tree, and other acetyltransferases were clustered with them. This result indicates that the *BEAT* genes in group I have far more unique copies in the *P. mume* genome than in *P. persica*. We inferred that the multiple copies of *PmBEATs* in group I may contribute to the production of benzyl acetate in the flowers of *P. mume*. Thus, we selected the 44 PmBEATs in group I for further study.

**Fig. 3:**
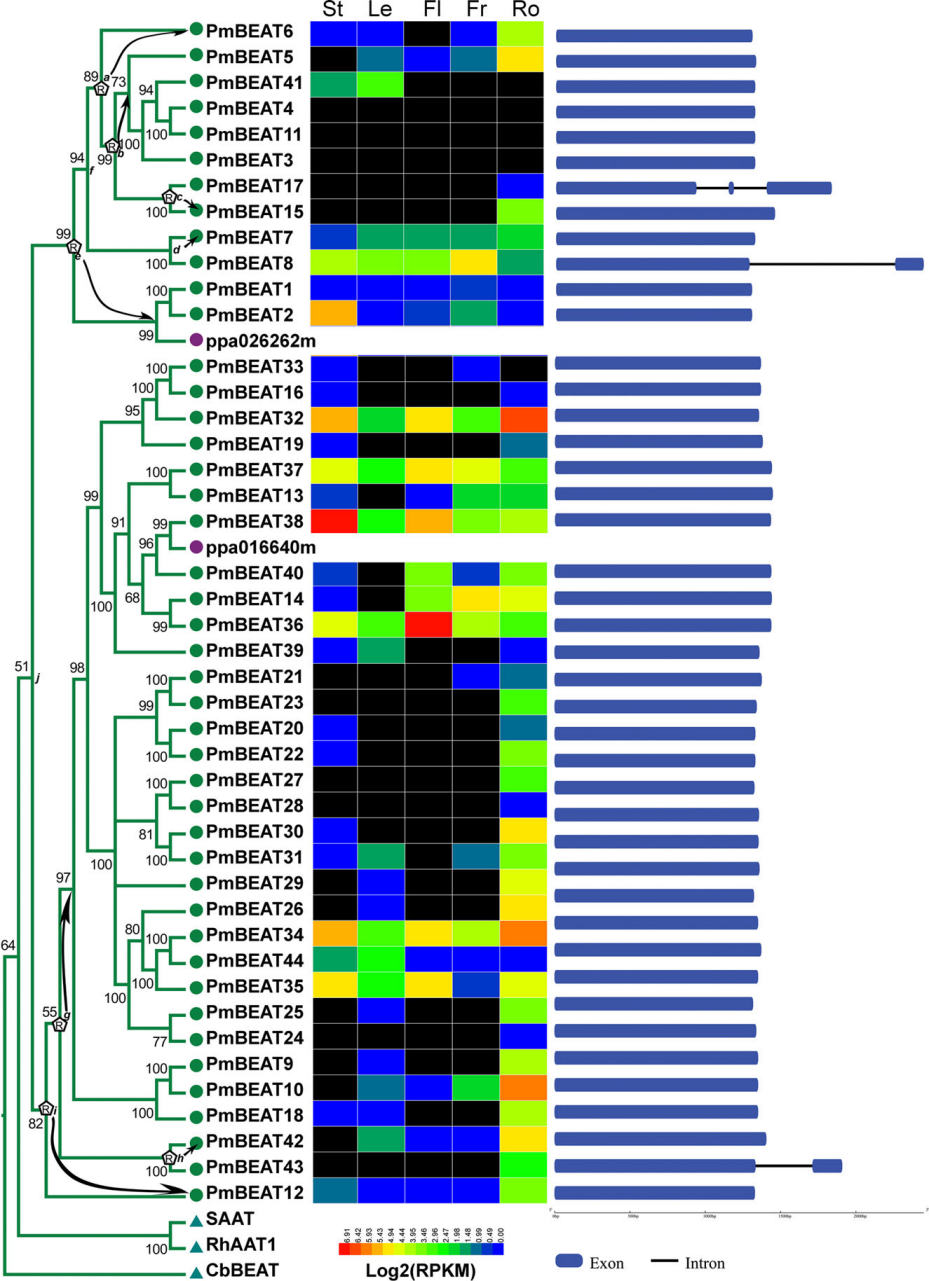
Gene retroduplication, structure and expression profiling of *PmBEAT* genes in different organs. The genes belonging to group I are listed. The evolutionary distances were computed using the Poisson correction method. The letter R on the nodes of the tree indicates the positions where retroduplication has occurred. Hypothesized donor genes and retrogenes in retroduplication events are connected by arrows. The colored boxes following the genes indicate their expression profiling. The color gradient from red to blue denotes expression levels from high to low. The genes with no transcription detected are in black. Five different organs are stems (St), leaves (Le), flowers (Fl), fruits (Fr), roots (Ro). In the gene structure analysis, the exons and introns are indicated by blue boxes and thin lines, respectively

Based on the transcriptome data of *P. mume*^24^, gene expression heatmaps were drawn to indicate the expression patterns of *PmBEATs* in different organs. The results show that different expression levels can be detected for 18 *PmBEAT* genes in the buds, 22 genes in the fruits, 26 genes in the stems, 24 genes in the leaves and 36 genes in the roots (Fig. 3). The *PmBEAT* family may play multiple roles in different organs. Gene structure analysis showed that the *PmBEAT* genes do not contain introns, with the exceptions of *PmBEAT17*, *PmBEAT8* and *PmBEAT43*.

### Location and duplication of *PmBEAT* genes in linkage groups

**Fig. 4:**
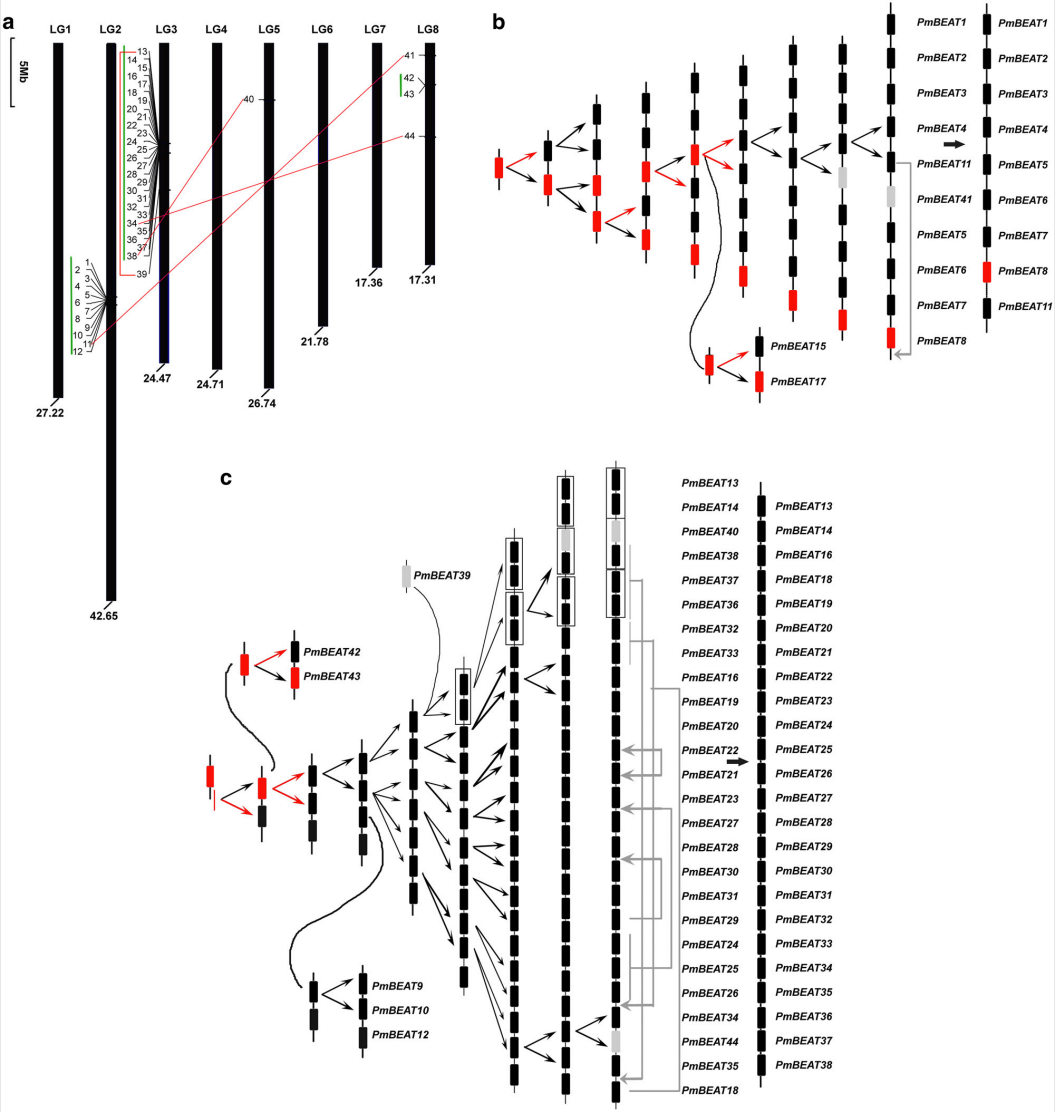
Linkage group locations and evolution of the *PmBEAT* genes in the genome of *P. mume*. **a** The 8 linkage groups correspond to 8 chromosomes of *P. mume*. The locations of *PmBEATs* are marked in the picture. The length of each chromosome in Mb is indicated below. The scale refers to a 5 Mb chromosomal distance. The segmentally duplicated genes are represented by red lines and tandemly duplicated genes by green vertical lines. The hypothetical origins of the *PmBEAT* genes in the clusters LG2 (**b**) and LG3 (**c**) formed by tandem duplications are shown. The retroduplication events and intron-containing genes are indicated by red arrows and red boxes, respectively. The segmentally duplicated genes are indicated in gray. Changes in gene positions caused by putative gene transposition or chromosome inversion events are indicated by gray arrows

Based on the genome database, we located 44 *PmBEAT* genes in group I in eight linkage groups, which correspond to eight chromosomes in *P. mume* (Fig. 4a). Tandem and segmental duplication events were analyzed to investigate the evolutionary processes of the *PmBEAT* family. Four segmental duplication events were identified in the *PmBEAT* family on the basis of the sequence similarity (Fig. 4a). Tandem duplication is the main evolutionary cause of expansion in the *PmBEAT* gene family. The distribution of the three clusters in LG2, LG3 and LG8 suggest that they originated from tandem duplication. We speculated on these tandem duplication processes based on the phylogenetic relationships among PmBEATs (Fig. 3). Phylogenetically, the genes *PmBEAT1*, *2*, *3*, *4*, *5*, *6*, *7*, *8* and *11* are in a single clade, suggesting that they may have resulted from tandem duplication and thus share the same ancestral gene (Fig. 4b). *PmBEAT15* and *PmBEAT17* in LG3 likely also originated from this ancestral gene. The largest *PmBEAT* gene cluster is located on LG3 and contains 24 tandem-arrayed members: *PmBEAT13*, *14*, *16*, *19*, *20*, *21*, *22*, *23*, *24*, *25*, *26*, *27*, *28*, *29*, *30*, *31*, *32*, *33*, *34*, *35*, *36*, *37* and *38*. However, these genes may be the result of more complex tandem duplication events. The clade they form also contains genes from other locations: *PmBEAT9*, *10* and *12* are located in the LG2 cluster, whereas *PmBEAT42* and *43* are located in LG8 (Fig. 4c). In addition, the ectopic recombination of chromosomes may have changed the locations of some genes in this gene cluster, including PmBEAT*36/37/38*, *32/33*, *21/22*, *24/25/26*, *29* and *18*. A similar event may have also occurred in *PmBEAT11* on LG2 (Fig. 4b). In addition, chromosome exchange between LG2 and LG3 may have changed the positions of *PmBEAT9*/*10*/*12* and *PmBEAT15*/*17*. Interestingly, we also found that the *PmBEAT12/13/36/27/38* cluster may have originated from a two-gene cluster through two tandem duplication events (Fig. 4c). The segmental duplication origins of *PmBEAT39*, *40*, *41* and *44* are also indicated by the putative duplication processes.

Since most of the *PmBEAT* genes lack introns, retroduplication may have occurred during gene expansion. Because a retrogene originates from the mRNA of a donor gene, the two have identical sequences except for the introns. Therefore, the retrogene will cluster together with the donor gene in the phylogenetic tree. Since *PmBEAT8*, *PmBEAT17* and *PmBEAT43* contain introns, possibly, nodes *d*, *c* and *h* of their recent ancestors also contain introns, as do nodes *a*, *b*, *e*, *f*, *g*, *i* and *j* of their elder ancestors. According to this speculation, the most likely locations of retroduplication events are in nodes *a*, *b*, *c*, *d*, *e*, *g*, *h* and *i* of the tree shown in Fig. 3, and the intron-containing genes are indicated in Fig. 4b, c. Additional evidence for the occurrence of retroduplication is that 12 of the *PmBEAT* genes have poly(A)-like tails in their 3′ flanking sequences; however, direct repeats cannot be identified in the 5′ and 3′ flanking sequences (Fig. S4). The poly(A) and short directed repeats of retrogenes very likely can be easily masked by base substitutions and/or insertions and deletions during the evolutionary process and thus may not be recognized^26^.

### Expression patterns of *PmBEAT* genes

To further study the spatiotemporal expression patterns of *PmBEAT* genes in the variety ‘Sanlunyudie’, all *PmBEAT* genes except *PmBEAT1, 3, 15, 28, 30, 42* and *43* were cloned from the experimental variety. The primers were designed according to the coding DNA sequences (CDS) from the genome database (Table S1). Some differences were observed at the nucleic acid and amino acid levels from the published sequences of *PmBEATs* from the wild plant used for genome sequencing. Remarkably, a fragment deletion in the coding sequence of *PmBEAT19* resulted in frameshifts with premature termination by introducing a stop codon (Fig. S2).

**Fig. 5:**
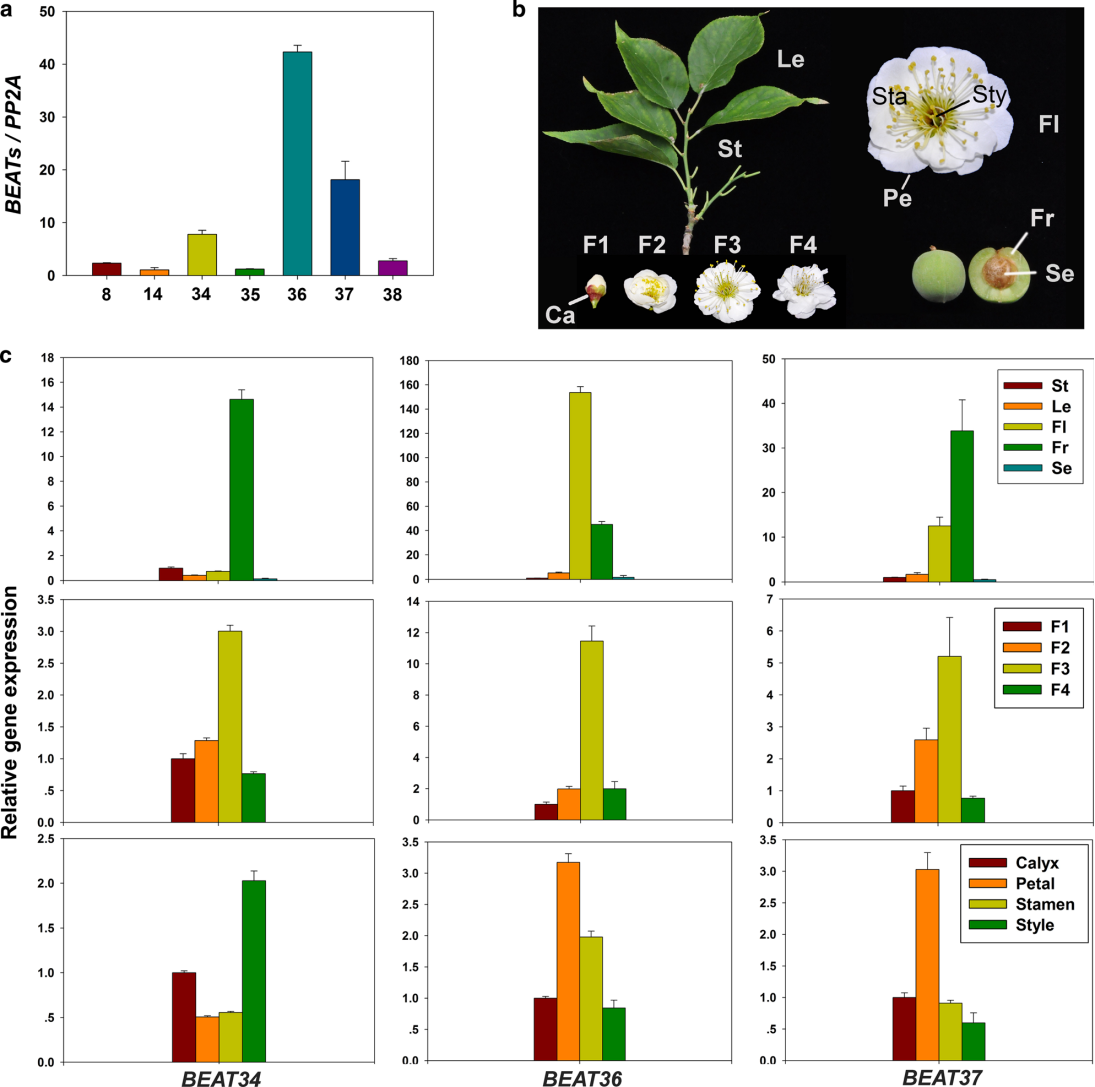
Expression patterns of *PmBEATs*. **a** The expression levels of *PmBEATs* in blooming flowers determined using real-time PCR. **b** The plant materials used for gene expression pattern analysis. St stems, Le leaves, Fl flowers, Fr fruits, Se seeds, F1 budding phase, F2 early flowering phase, F3 blooming phase, F4 late flowering phase. A blooming flower was dissected into four different tissues, the calyx (Ca), petal (Pe), stamen (Sta) and style (Sty) to determine the gene expression. **c** The expression patterns of *PmBEAT34*, *PmBEAT36* and *PmBEAT37* in different organs, flowering stages and flower parts. The gene *PmPP2A* was used as a reference gene. The data are presented as the mean values of three replicates ± SD. Three independent experiments were performed with similar results

The endogenous synthesis of benzyl acetate has been reported to occur mainly in petals and stamens, with the benzyl acetate content reaching its peak during the blooming stage. We asked whether the expression patterns of *PmBEATs* are consistent with this pattern of endogenous benzyl acetate synthesis. To identify the key genes that control fragrance synthesis in the flowers, we determined the expression levels of *PmBEATs* in five different tissues of *P. mume* using semiquantitative reverse transcription polymerase chain reaction (RT-PCR; Fig. S3). The primers were designed according to the coding sequences of *PmBEATs* in the ‘*Sanlunyudie*’ variety (Table S2). We detected *PmBEAT8*, *PmBEAT14*, *PmBEAT34*, *PmBEAT35*, *PmBEAT36*, *PmBEAT37* and *PmBEAT38* in the flower tissues. This result is generally consistent with the transcriptome data. Next, the expression levels of these *PmBEAT* genes in the flowers were compared using real-time PCR (the primers are listed in Table S3). As shown in Fig. 5a, the expression levels of *PmBEAT34*, *PmBEAT36* and *PmBEAT37* were more than three times greater than the expression levels of other genes in the flowers of *P. mume*. We speculate that *PmBEAT34*, *PmBEAT36* and *PmBEAT37* may play the most important role in benzyl acetate synthesis. The results of an analysis of the temporal and spatial expression patterns of these genes (Fig. 5b) show that *PmBEAT34* was mainly expressed in fruits and had relatively high expression levels in the styles. By contrast, *PmBEAT36* was mostly expressed in flowers and fruits, but the highest levels of expression were in the flowers, particularly in the petals and stamens. Finally, *PmBEAT37* was also mainly expressed in flowers and fruits, with high expression levels in fruits and the highest expression level in the petals (Fig. 5c). The flowering process was divided into four stages: F1 (the budding phase, before the flowers opened), F2 (the early flowering phase, when the flowers began to open but were not fully expanded), F3 (the blooming phase, when the flowers were unfolded with yellow stamens) and F4 (the late flowering phase, when the petals began to shed, and the stamens faded) (Fig. 5b). The expression levels of *PmBEAT34*, *PmBEAT36* and *PmBEAT37* all increased throughout the process of flowering, peaked during the blooming stage and then decreased during the late flowering stage (Fig. 5c). In addition, the expression levels of *PpBEAT21* and *PpBEAT35* in three tissues of *P. persica* were analyzed. As shown in Fig. S3, they were mainly expressed in leaves and stems and could not be detected in flowers.

### Conserved motif analysis of the PmBEATs

**Fig. 6:**
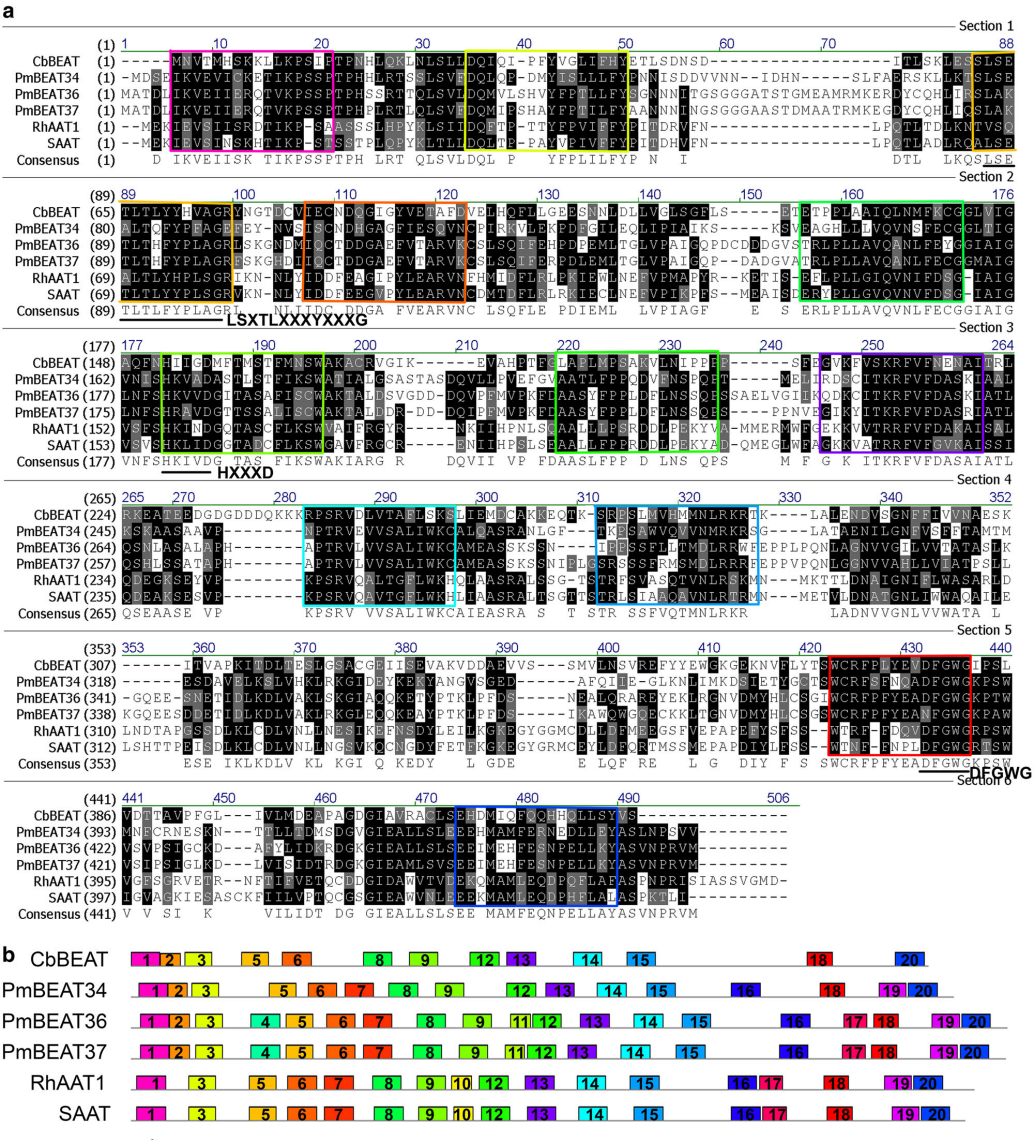
Comparison of the amino acid sequences and motifs of PmBEATs with those of closely related alcohol acetyltransferases from other species. **a** Comparison of the amino acid sequences of alcohol acetyltransferases. The conserved motifs are shown in colored squares; different colors reflect the different motifs in (**b**), and the reported conserved motifs are indicated with black lines. **b** Comparison of the motifs of alcohol acetyltransferases. Each motif is represented by a colored block with a number. The lengths and positions of the blocks correspond to the lengths and positions of the motifs in the individual protein sequences

We then analyzed the conserved motifs of the PmBEAT34, PmBEAT36 and PmBEAT37 proteins. Their amino acid sequences were compared to those of the CbBEAT, RhAAT1 and SAAT proteins. The results show that the three PmBEAT proteins shared sequence identities of 24–28% with CbBEAT. Although the homology of the amino acid sequences was low, the protein motif analysis showed 12 consensus sequences, including the published H*XXX*D, DFGWG and LS*X*TL*XXX*Y*XXX*G motifs (*X* is a variable-identity amino acid) (Fig. 6a). H*XXX*D and DFGWG have been reported to be the two most highly conserved motifs in CoA-dependent acyltransferases^13^. The H*XXX*D motif is located in the center of the reaction channel of the protein. The DFGWG motif near the C terminus of the protein is considered to be indispensable for catalysis. The LS*X*TL*XXX*Y*XXX*G motif, located near the N terminus, may play an important role in reactions that use acetyl-CoA as a substrate^3^. To identify the conserved motifs and evaluate the structural divergences of the PmBEATs, the MEME online tool was used to analyze the motif compositions compared to those of other reported alcohol acetyltransferases. Figure 6b shows a model diagram of the distribution of conserved motifs. In total, 12 conserved motifs were identified in all the sequences: motifs 1, 3, 5, 6, 8, 9, 12, 13, 14, 15, 18 and 20. Motifs 5, 9 and 18 correspond to the LS*X*TL*XXX*Y*XXX*G, H*XXX*D and DFGWG motifs, respectively (Fig. 6a). Other motifs were found in some sequences but not in others. Three motifs (7, 16 and 19) were shared in all sequences except that of the CbBEAT protein, whereas motif 2 was specifically identified in CbBEAT and the PmBEATs. Two motifs, 4 and 11, were present only in PmBEAT36 and PmBEAT37, whereas motif 10 was unique to the RhAAT1 and SAAT proteins. In addition, motif 17 was identified only in the PmBEAT36, PmBEAT37, RhAAT1 and SAAT proteins.

### Enzyme activities of the PmBEAT proteins

**Fig. 7:**
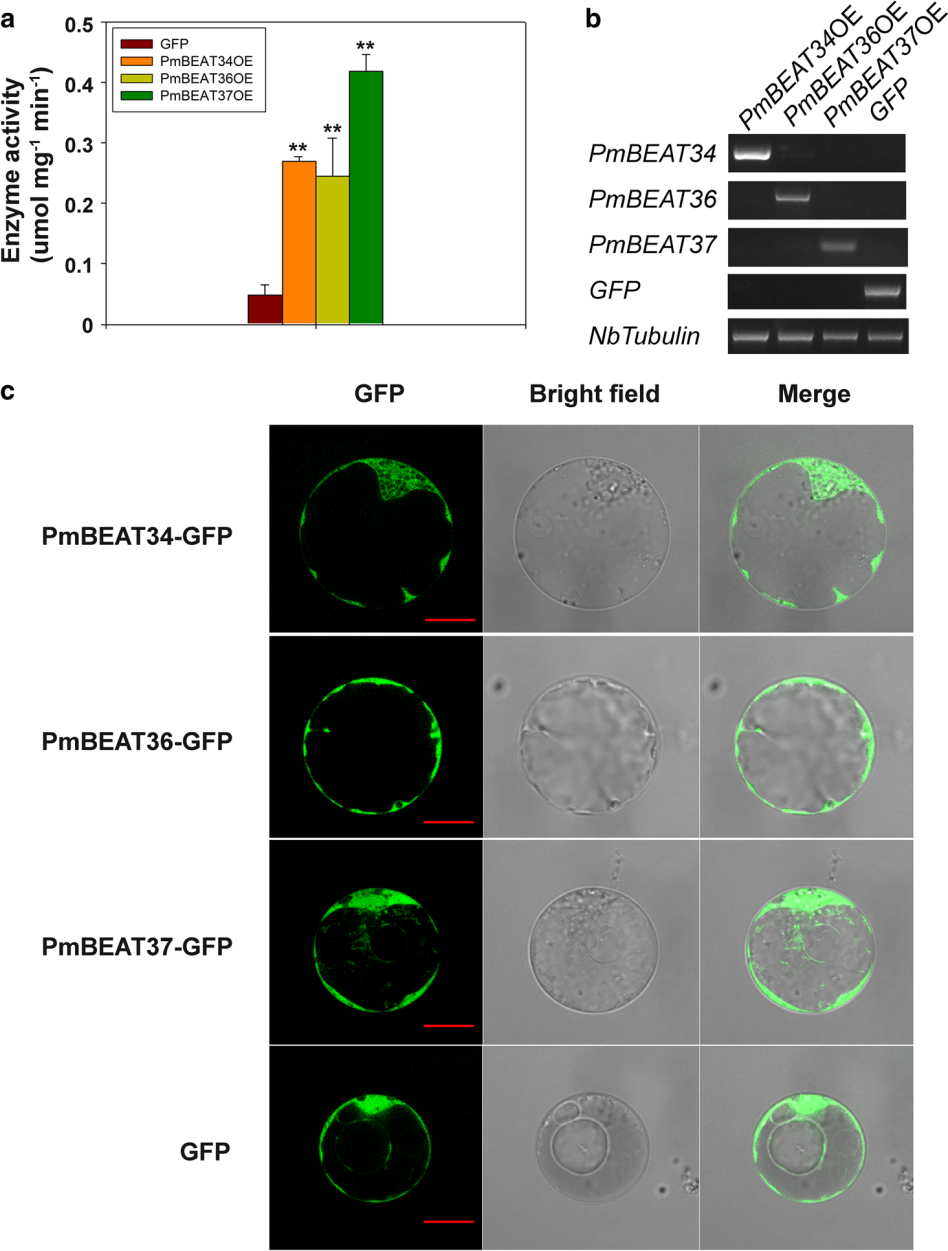
Enzyme activity and subcellular localization analysis of PmBEATs. **a** The benzyl alcohol acetyltransferase activity of PmBEAT34, PmBEAT36 and PmBEAT37. The *PmBEAT* genes were expressed in tobacco leaves by *Agrobacterium* injection, and the crude protein was extracted for enzyme activity analysis. The data are presented as the mean values of three replicates ± SD. **P < 0.01 (Student’s *t*-test). Three independent experiments were performed with similar results. **b** Detection of the expression levels of *PmBEAT34*, *PmBEAT36* and *PmBEAT37* in tobacco leaves by sq-RT-PCR. *NbTubulin* was used as the reference gene. **c** Confocal images of petal protoplasts expressing PmBEAT proteins containing the GFP tag. The scale bars are 20 μM

To verify the benzyl alcohol acetyltransferase activities of PmBEATs, *PmBEAT34*, *PmBEAT36* and *PmBEAT37* were heterologously expressed in the leaves of tobacco plants, and the crude proteins were extracted to determine the enzyme activities of PmBEATs in in vitro systems. The hydrosulfonyl produced from acetyl-CoA was used to calculate the enzyme activity using 5,5′-dithiobis-(2-nitrobenzoic acid) (DTNB) with a spectrophotometer at a wavelength of 412 nm. As shown in Fig. 7a, significant enzyme activities were detected in the leaves, which overexpressed *PmBEAT34*, *PmBEAT36* and *PmBEAT37* compared with the control. In addition, the activity of PmBEAT37 was higher than that of PmBEAT34 and PmBEAT36. The gene expression levels in tobacco leaves were detected using RT-PCR (Fig. 7b). To further assess the location at which benzyl acetate was synthesized in the cell, constructs with PmBEATs infused with green fluorescent protein (GFP) were introduced into the petal protoplasts of *P. mume* to determine the subcellular localizations. As shown in Fig. 7c, PmBEAT34, PmBEAT36 and PmBEAT37 were all localized in the cytoplasm. These results indicate that benzyl acetate was synthesized in the cytoplasm of petal cells.

### *PmBEATs* affect the synthesis of benzyl acetate in the petal cells of *P. mume*

**Fig. 8:**
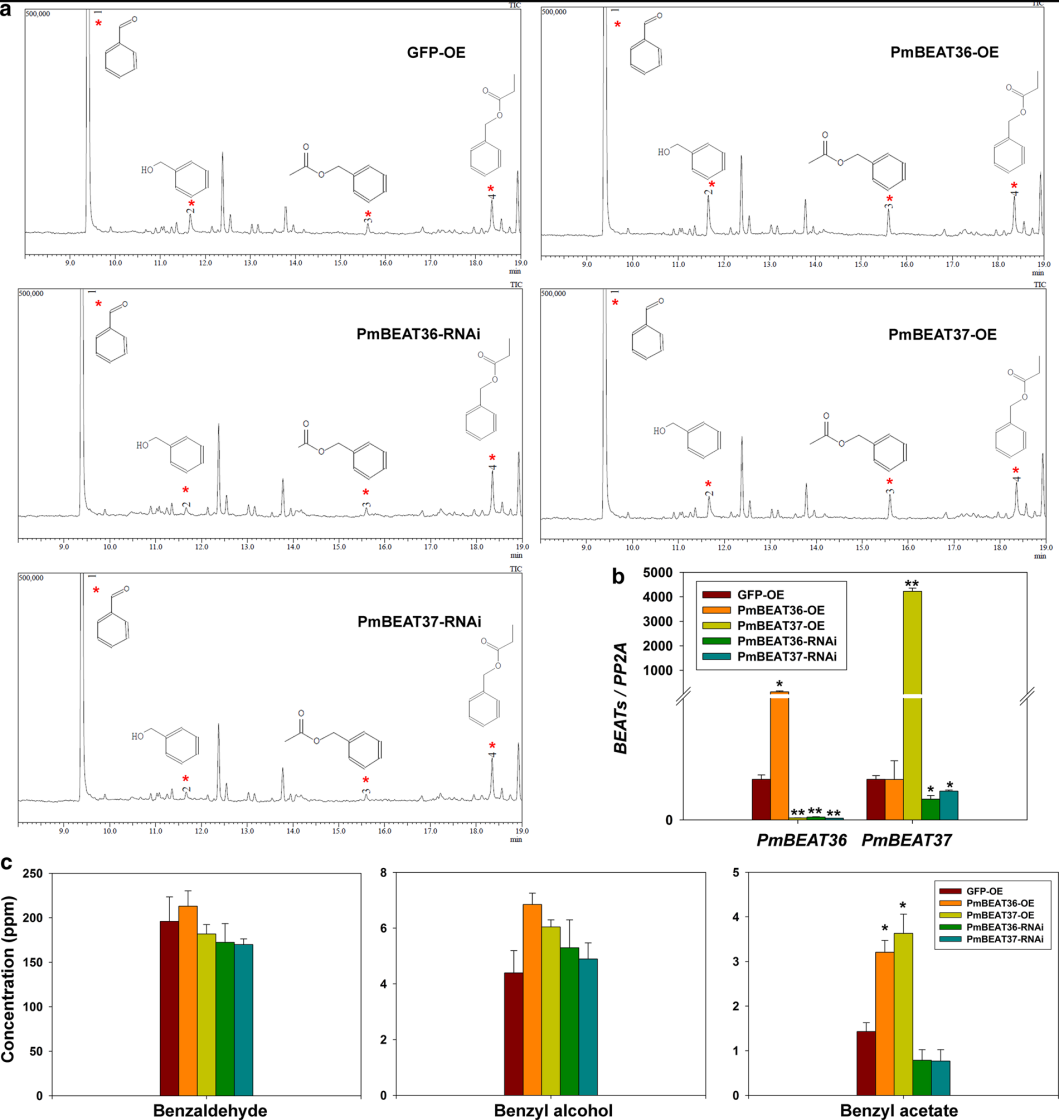
Expression levels of *PmBEATs* affect the benzyl acetate content in the petal protoplasts of *P. mume*. **a** Petal protoplasts transfected with *PmBEAT* overexpression or RNA-interference constructs were collected to determine the three components of the floral fragrance using GC-MS. The peaks corresponding to benzaldehyde, benzyl alcohol and benzyl acetate are indicated with red asterisks. The sample overexpressing the GFP was used as a control. **b** The expression levels of *PmBEAT36* and *PmBEAT37* in protoplasts were analyzed by real-time PCR. **c** Quantitative analysis of benzaldehyde, benzyl alcohol and benzyl acetate in protoplasts. The data are presented as the mean values of three replicates ± SD. *P < 0.05 and **P < 0.01 (Student’s *t*-test). Three independent experiments were performed with similar results

The petals were the main tissue in which benzyl acetate synthesis occurred in *P. mume*. Because mature genetic transformation and regeneration systems were not available, the protoplast transient transformation system was used to verify the functioning of PmBEATs in vivo. Since *PmBEAT34* was mainly expressed in the fruits, and *PmBEAT36* and *PmBEAT37* were mainly expressed in the flowers and fruits and had the highest expression levels in petal tissue, *PmBEAT36* and *PmBEAT37* were selected for functional verification in the petal protoplasts. The petal protoplasts were prepared and introduced with overexpressing or interfering gene constructions. After incubation at 20 °C for 30 h, the protoplasts were collected for endogenous component extraction, and the contents of benzaldehyde, benzyl alcohol and benzyl acetate were then detected using gas chromatography-mass spectrometry (GC-MS; Fig. 8a). The RNA was also extracted for gene expression analysis. As shown in Fig. 8b, the expression levels of *PmBEAT36* and *PmBEAT37* were significantly increased in the protoplasts of samples overexpressing those *PmBEATs* and were reduced in the interfering expression samples. We note that the overexpression of *PmBEAT37* also inhibited the expression of *PmBEAT36* in protoplasts. As shown in Fig. 8c, the contents of benzaldehyde, benzyl alcohol and benzyl acetate were calibrated using an internal standard. The levels of benzyl acetate increased significantly when *PmBEAT36* or *PmBEAT37* was overexpressed, and decreased slightly in the interfering expression samples compared with the control. These results indicate that the expression levels of *PmBEAT36* or *PmBEAT37* can affect the synthesis of benzyl acetate in the flower cells of *P. mume*. In addition, we found that the content of benzyl alcohol was slightly elevated when *PmBEAT36* or *PmBEAT37* was overexpressed in cells, whereas no obvious differences were observed in the benzaldehyde content.

### Light and temperature can affect the expression of the *PmBEATs*

**Fig. 9:**
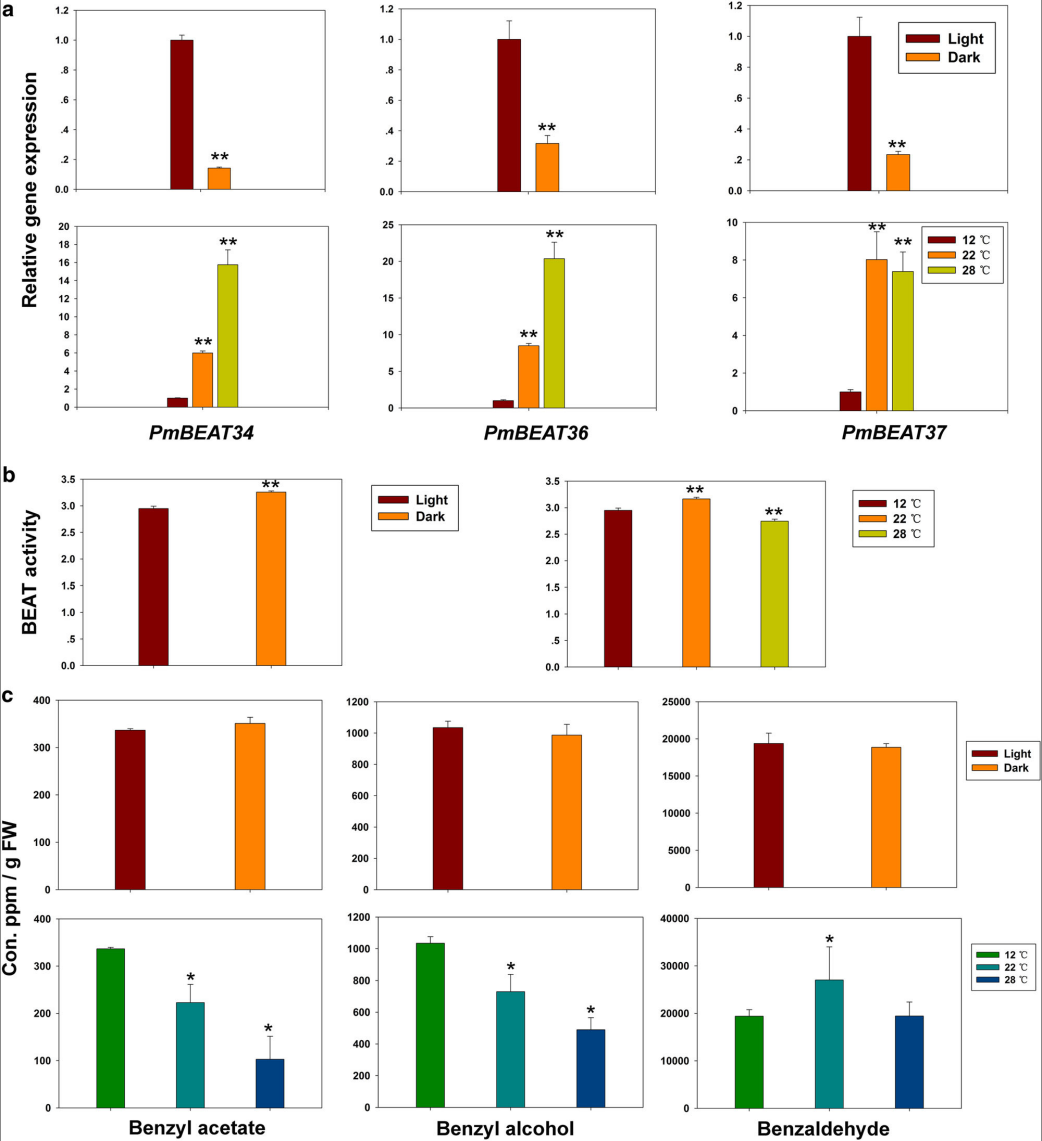
Light and temperature affect the benzyl acetate synthesis pathway. (**a**) The expression levels of *PmBEAT34*, *PmBEAT36* and *PmBEAT37*, (**b**) the benzyl alcohol acetyltransferase activity and (**c**) the endogenous contents of benzaldehyde, benzyl alcohol and benzyl acetate in blooming flowers under different light or temperature treatments. The data are presented as the mean values of three replicates ± SD. * indicates P < 0.05, and ** indicates P < 0.01 (Student’s *t*-test). Three independent experiments were performed with similar results

To further study the regulatory mechanisms of *PmBEAT* gene expression, the promoter sequences of *PmBEAT34*, *PmBEAT36* and *PmBEAT37* were obtained from the genome database of *P. mume*, and a search for *cis*-acting regulatory elements was performed using the PlantCARE online tool. Several light- or temperature-responsive elements were identified in the promoter sequences of *PmBEAT* genes (Table 1). To examine how light affected the expression of the *PmBEATs*, detached branches with buds were dipped in water and cultured in a 12 °C incubator with or without light. When the buds were blooming, the flowers were collected for gene expression analysis. As shown in Fig. 9a, the gene expression levels of *PmBEATs* were higher in the flowers that opened in the light than in those that opened in the dark. These results indicate that light positively regulates the *PmBEAT* gene expression. The benzyl alcohol acetyltransferase activity and endogenous fragrance content were also analyzed in flowers that opened in the light versus the dark. Interestingly, the enzyme activity significantly increased when the flowers opened in the dark (Fig. 9b), but no differences were observed in the contents of benzyl acetate, benzyl alcohol and benzaldehyde in the light versus dark treatments (Fig. 9c). Possibly, a post-transcriptional regulation mechanism exists that affects the enzyme activity, and thus the benzyl alcohol content may be a more important limiting factor in the synthesis of benzyl acetate.

**Table 1:** The light- and temperature-responsive elements in the promoters of *PmBEAT* genes

Gene	Site name	Sequence	Number	Function
*PmBEAT34*	5' UTR Py-rich stretch	TTTCTTCTCT	1	*Cis*-acting element conferring high transcription levels
		TTTCTCTCTCTCTC	1	
	AE-box	AGAAACAA	1	Part of a module for light response
	Box 4	ATTAAT	1	Part of a conserved DNA module involved in light responsiveness
	G-box	CACGTG	1	*Cis*-acting regulatory element involved in light responsiveness
		CACACATGGAA	1	
		CACGTA	1	
		TACGTG	1	
	GA-motif	AAAGATGA	1	Part of a light-responsive element
	GT1-motif	GGTTAA	1	Light-responsive element
		AATCCACA	1	
	Gap-box	AAATGGAGA	1	Part of a light-responsive element
	L-box	ATCCCACCTAC	1	Part of a light-responsive element
	LTR	CCGAAA	1	*Cis*-acting element involved in low-temperature responsiveness
	Sp1	CC(G/A)CCC	1	Light-responsive element
	TCT-motif	TCTTAC	2	Part of a light-responsive element
	chs-CMA1a	TTACTTAA	1	Part of a light-responsive element
	rbcS-CMA7a	GTCGATAAGG	1	Part of a light-responsive element
*PmBEAT36*	AE-box	AGAAACAA	2	Part of a module for light response
	Box 4	ATTAAT	2	Part of a conserved DNA module involved in light responsiveness
	G-box	CACGAC	1	*Cis*-acting regulatory element involved in light responsiveness
		GACACGTAGT	1	
	GTGGC-motif	GATTCTGTGGC	1	Part of a light-responsive element
	HSE	AAAAAATTTC	3	*Cis*-acting element involved in heat-stress responsiveness
		AGAAAATTCG	1	
	L-box	ATCCCACCTAC	1	Part of a light-responsive element
	Sp1	GGGCGG	1	Light-responsive element
	TCT-motif	TCTTAC	2	Part of a light-responsive element
*PmBEAT37*	AT1-motif	AATTATTTTTTATT	1	Part of a light-responsive module
	ATCT-motif	AATCTGATCG	1	Part of a conserved DNA module involved in light responsiveness
	Box 4	ATTAAT	2	Part of a conserved DNA module involved in light responsiveness
	Box I	TTTCAAA	2	Light-responsive element
	CATT-motif	GCATTC	1	Part of a light-responsive element
	G-box	CACGAC	1	*Cis*-acting regulatory element involved in light responsiveness
	GAG-motif	GGAGATG	1	Part of a light-responsive element
		AGAGAGT	2	
*PmBEAT37*	GATA-motif	GATAGGG	1	Part of a light-responsive element
	HSE	AAAAAATTTC	1	*Cis*-acting element involved in heat-stress responsiveness
	I-box	GATAGGG	1	Part of a light-responsive element
	L-box	ATCCCACCTAC	1	Part of a light-responsive element
	LTR	CCGAAA	2	*Cis*-acting element involved in low-temperature responsiveness
	Sp1	CC(G/A)CCC	4	Light-responsive element
	TCT-motif	TCTTAC	3	Part of a light-responsive element
	chs-CMA1a	TTACTTAA	2	Part of a light-responsive element

To assess how temperature affects the synthesis of benzyl acetate, detached branches were treated in lighted incubators at 12 °C, 22 °C and 28 °C. Once the buds were blooming, the flowers were analyzed for gene expression, enzyme activity and endogenous fragrance content. As shown in Fig. 9a, the gene expression levels of *PmBEATs* increased significantly with temperature. However, the benzyl alcohol acetyltransferase activity was increased at 22 °C and decreased at 28 °C compared to that at 12 °C (Fig. 9b). The inconsistency between gene expression and enzyme activity also implies the existence of post-transcriptional regulation of enzyme activity. By contrast, the contents of benzyl acetate and benzyl alcohol decreased as the temperature increased (Fig. 9c). In addition, the benzaldehyde content increased at 22 °C, but no significant difference was observed at 28 °C compared to the treatment at 12 °C. The trend in endogenous benzyl acetate content was consistent with the benzyl alcohol substrate, which might suggest that the content of benzyl alcohol is the most important factor affecting benzyl acetate synthesis at different temperatures.

## Discussion

**Fig. 10:**
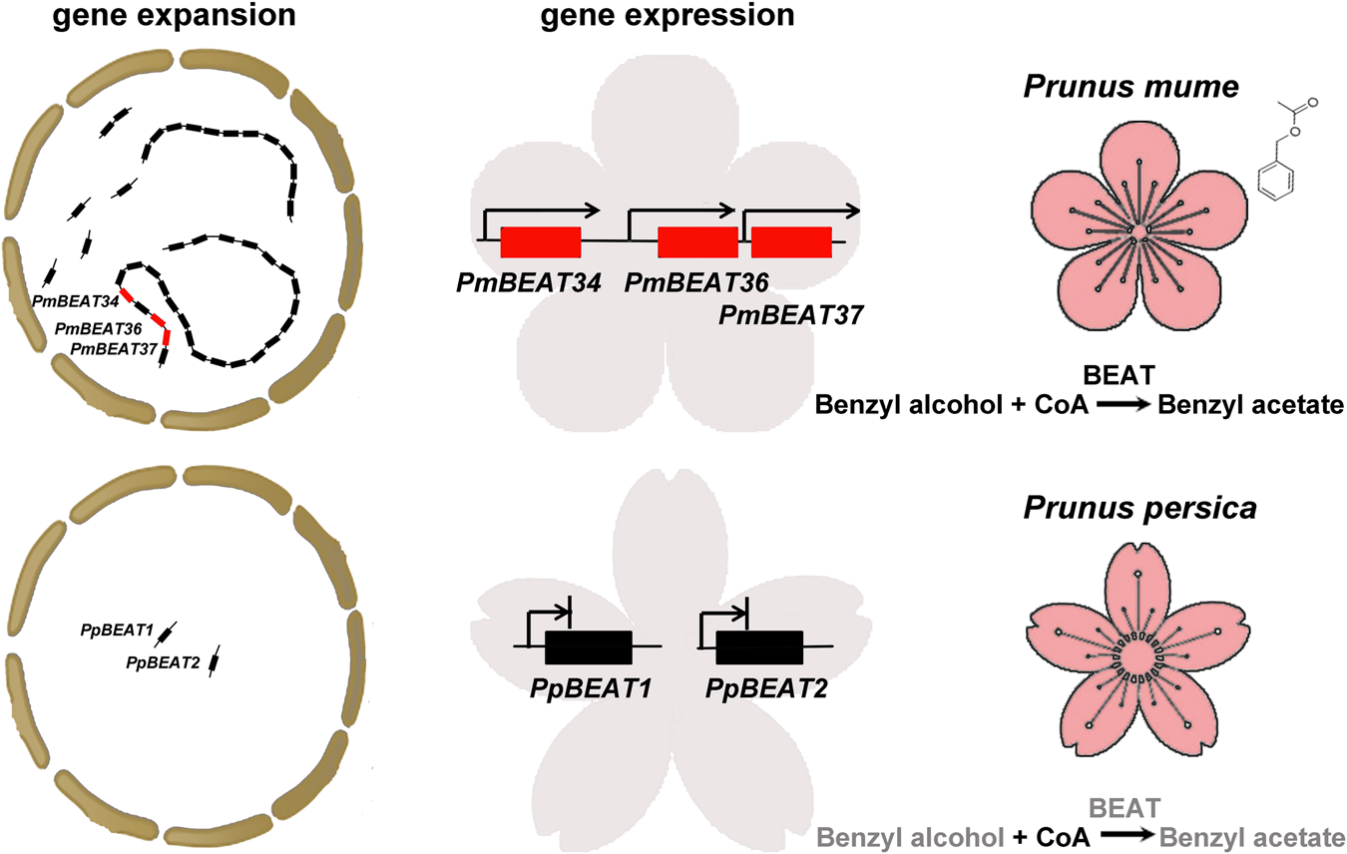
Model for *PmBEAT* gene evolution and function in the flowers of *P. mume*. Two copies of *PpBEAT* genes exist in the genome of *P. persica*, but the expression of 2 *PpBEATs* genes was not observed in the flowers. Forty-four copies of *PmBEAT* genes were produced through gene expansion, and the expression of *PmBEAT34*, *PmBEAT36* and *PmBEAT37*, which were generated by tandem duplications, were actively observed in the flowers and resulted in the high benzyl alcohol acetyltransferase activity observed in *P. mume*. In addition, benzyl alcohol can be synthesized in *P. mume*, but not in *P. persica*. Therefore, benzyl acetate, which is the main component of characteristic floral scent, can be synthesized in *P. mume*

An examination of the distribution of the *BEAT* genes in the *P. mume* and *P. persica* genomes reveals many more *PmBEAT* genes in the *P. mume* genome. In group I, 44 *PmBEAT* genes were extracted from the *P. mume* genome, whereas 2 *BEAT* genes were extracted from the *P. persica* genome. This result indicates that the duplication and evolution events of the *PmBEAT* genes in the *P. mume* genome occurred after the differentiation of the two species (*P. mume* and *P*. *persica*). The 44 *PmBEATs* and 2 *PpBEATs* may share a common ancestral gene and a similar function. Our study showed that tandem duplication and retroduplication played dominant roles in the expansion of the *PmBEAT* gene family. The retroduplication events occurred earlier than the tandem duplication events. Most retrogenes are generally believed to be nonfunctional because of the absence of regulatory sequences, and retroduplication is believed to play a very minor role in the expansion of gene families. In the *PmBEAT* gene family, however, most of the members, except *PmBEAT3*, *PmBEAT4* and *PmBEAT11*, have been detected in transcription data, indicating that they may be functional. *PmBEAT34, PmBEAT36 and PmBEAT37* are generated by tandem duplication, and an examination of their enzyme activities in vitro and in vivo has indicated that the *PmBEAT* genes are functional. *P. mume* has higher benzyl alcohol acetyltransferase activity than *P. persica* and can synthesize benzyl acetate, which is manifested in the fragrance of the flowers and fruits of *P. mume*. The multiple copies of the *PmBEAT* genes play an important role in the characteristic aroma of *P. mume*. The expression of the two *PpBEAT* genes was detected only in leaves and stems but not in the peach flowers. We hypothesize that the expansion of the *PmBEAT* genes coupled with varied gene expression patterns resulted in specific expression in flowers and thereby induced the heightened activity of the benzyl alcohol acetyltransferase, which resulted in the synthesis of benzyl acetate in the flowers of *P. mume* (Fig. 10). The numerous repeats of the *BEAT* genes clustered in the genome are unique and have not been observed in other species. Our study indicates that the expansion of the *PmBEAT* genes in the genome resulted in the unique species characteristics of *P. mume* and presents new evidence that retroduplication can play an important role in the formation of new metabolic pathways.

In addition to the presence of the *BEAT* genes in the genome, the synthesis of benzyl acetate also requires sufficient benzyl alcohol and acetyl-CoA substrates. Acetyl-CoA is abundant in the cytoplast of plant cells. The characteristic fragrance of *P. mume* flowers is due to the benzyl alcohol synthesis pathway and to benzyl alcohol acetyltransferase. The benzyl alcohol acetyltransferase activity and the benzyl alcohol content limit the synthesis of benzyl acetate in *P. mume*. Our results suggest that benzyl alcohol acetyltransferase activity in flowers is regulated not only by the transcription levels of the *PmBEAT* genes but more importantly by post-transcriptional mechanisms. Although light can increase the expression of *PmBEAT* genes, it results in decreased benzyl alcohol acetyltransferase activity in the flowers and has no significant effect on the synthesis of endogenous benzyl acetate and benzyl alcohol. Temperature, in contrast, has considerable influence on the synthesis of endogenous benzyl alcohol and benzyl acetate. Higher temperatures can increase the expression levels of *PmBEATs* and the activity of benzyl alcohol acetyltransferase in flowers, but decrease the synthesis of the benzyl alcohol substrate, which restricts the synthesis of benzyl acetate and, ultimately, leads to a decrease in the content of endogenous benzyl acetate. In contrast to the results of the study on *P. mume*, neither benzyl alcohol nor benzyl alcohol acetyltransferase activity was detected in *P. persica*, indicating that no relevant metabolic pathway exists for the synthesis of benzyl acetate in the flowers of *P. persica*. Studies using the isotope tracing method in petunia reported that benzyl alcohol is produced by the reduction of benzaldehyde and is reversible^10^. Thus far, no enzyme has been identified that can synthesize benzyl alcohol in plants. We will focus on this question in future research.

The results of this study showed that *PmBEAT34*, *PmBEAT36* and *PmBEAT37* were highly expressed in floral organs, and they were verified to have benzyl alcohol acetyltransferase activities in vitro. However, the enzyme activities were tested using the crude extracts, which could only indirectly show that the candidate genes can enhance the benzyl alcohol acetyltransferase activity. To further study the specific enzyme activities of PmBEATs, the background interference should be eliminated. *PmBEAT36* and *PmBEAT37* are mainly expressed in petal tissues. Manipulating the expression levels of *PmBEAT36* and *PmBEAT37* in the petal protoplasts of *P. mume* affected the endogenous benzyl acetate content. We conclude that *PmBEAT36* and *PmBEAT37* are the major genes responsible for the synthesis of benzyl acetate in flowers. By contrast, *PmBEAT34* is mainly expressed in fruits and may play a major role in fruit flavor. In addition, although some of the *PmBEAT* gene family members are not expressed as strongly as *PmBEAT34*, *PmBEAT36* and *PmBEAT37* in the floral organs, they may also participate in the synthesis of benzyl acetate in the flowers. The *PmBEAT* gene copy number in the *P. mume* genome is high, and the gene expression patterns are varied. Some genes were detected only in the roots, whereas some genes were not detected in the transcriptome data of the various organs at all. We speculate that their expression may be induced by certain conditions. Therefore, we suggest that the function of the *PmBEAT* gene family is not limited to the synthesis of benzyl acetate in flowers and fruits; rather, these genes may have additional functions in *P. mume*.

## Materials and methods

### Genomic data mining and PmBEAT identification

To identify PmBEATs in *P. mume*, local BLASTP searches in the *P. mume* genome database were performed using the CbBEAT full-length protein sequence as the reference sequence. The PpBEATs were obtained using the same method in the *P. persica* genome database. The gene IDs are shown in Table S4. The PmBEAT and PpBEAT protein sequences were aligned with those of published acyltransferase, namely CbBEAT (AAC18062), CbBEBT (AAN09796), RhAAT1 (AAW31948), SAAT (AAG13130), PhCFAT (DQ767969), PhBPBT (AAU06226), MpAAT1 (AAU14879), AMAT (AAW22989), PtACT47 (KP228018), PtACT49 (KP228019) and AtCHAT (AAN09797), using ClustalW. The evolutionary history was inferred using the neighbor-joining method^27^. The phylogenetic tree of these BEAT proteins and the alcohol acetyltransferases reported in other species was created in MEGA6^28^. The evolutionary distances were computed using the Poisson correction method. A heat map was drawn according to the transcription data from the different organs of *P. mume*. The diagrams of gene structures were drawn based on *P. mume* sequencing data.

### Physical locations and gene duplications of *PmBEAT* genes

To determine the physical locations of *PmBEAT* genes, the starting and ending positions of the *PmBEAT* genes in each linkage group were obtained from the genome database of *P. mume*. The tandem duplications were identified based on the close phylogenetic relationship between tandemly arrayed genes at the same chromosomal location^20^. To determine the gene segmental duplications, the CDSs of *PmBEAT* genes were blasted against each other (e value < 1e–10, identity >80%), and the genes with the highest identity value were found. To examine whether *PmBEAT* genes are retrogenes, 1 kb noncoding sequences in the 5′ and 3′ ends of the genes were extracted from the genome sequence to identify the poly(A) tails and direct repeats.

### Gene expression pattern analysis

The primers were designed according to the CDS from the genome database to amplify the predicted *PmBEAT* genes from the genomic DNA and mRNA of ‘Sanlunyudie’. The predicted genes were then cloned into the pCloneEZ vector and then sequenced. The primers used in gene amplification are shown in Supplemental Table 1. The primers used for semiquantitative RT-PCR and real-time PCR were designed according to the coding sequences of the *PmBEAT* genes of ‘Sanlunyudie’. *PmPP2A* was used as a reference gene. The stems, leaves, flowers, fruits and seeds of ‘Sanlunyudie’ were collected for semiquantitative RT-PCR and real-time PCR to determine the gene expression in the different tissues. Flowers in four different stages during the open period and four different tissues from the blooming flowers were collected for real-time PCR to detect the temporal and spatial expression patterns of the genes. The primers used to detect *PpBEAT* gene expression are shown in Supplemental Table S2.

### Amino acid sequencing and conserved motif analysis

The amino acid sequences of alcohol acetyltransferases were aligned using Vector NTI software. The MEME online tool (http://meme-suite.org) was used for conserved motif analysis with the following parameters: the maximum number of motifs was 20, the minimum motif width was 6 and the maximum motif width was 16^29^.

### Extraction and analysis of the endogenous floral components of flowers

Fresh flowers in bloom were collected and ground into powder in liquid nitrogen. Approximately 0.2 g of powder was placed in a 2 mL centrifuge tube, and 1 mL ethyl acetate was added. The floral components were extracted on a vortex mixer for 15 min and then centrifuged at 12,000 rpm for 10 min. The supernatant was transferred to a new centrifuge tube, and anhydrous sodium sulfate was added to remove water. Benzyl propionate was added as an internal standard^1^. The components of the extracts were analyzed using GC-MS (GPC-GC, Shimadzu, Japan).

### Plasmid construction

To detect the activities of PmBEAT proteins and verify their functions, the CDS sequences of *PmBEAT34* (MH259759), *PmBEAT36* (MH259760) and *PmBEAT37* (MH259761) were cloned into the vector pSuper1300^30^ with a Super promoter, using the In-Fusion method (In-Fusion HD Cloning Kit, PT5162-1). The *PmBEAT34* complementary DN (cDNA) was amplified using the PmBEAT34OE-F (ACATTTAAATACTAGTatggattcagaaatcaaag) and PmBEAT34OE-R (TACCGGATCCACTAGTttaaacgacactcggattc) primers. The *PmBEAT36* cDNA was amplified using the PmBEAT36OE-F (ACATTTAAATACTAGTatggccacagacttgatc) and PmBEAT36OE-R (TACCGGATCCACTAGTatgacccttggatttac) primers. The *PmBEAT37* cDNA was amplified using the PmBEAT37OE-F (ACATTTAAATACTAGTatggccacagacttgatca) and PmBEAT37OE-R (TACCGGATCCACTAGTctacatgacccttggattta) primers.

To verify the functions of the *PmBEAT36* and *PmBEAT37* genes, specific fragments of the genes were cloned into the pFGC5941 vector. The two specific fragments of the *PmBEAT36* gene were amplified using the PmBEAT36-RNAi-F1 (tttacaattaccatggcaaacataccaccatcttcg) and PmBEAT36-RNAi-R1 (tgggcgcgccccatggtgcaatacctttactgagtttgg) primers and the PmBEAT36-RNAi-F2 (gcaggtatttggatcctgcaatacctttactgagtttgg) and PmBEAT36-RNAi-R2 (gactcacctaggatcccaaacataccaccatcttcg) primers, respectively. The specific fragments of the *PmBEAT37* gene were amplified using the PmBEAT37-RNAi-F1 (tttacaattaCCATGGgatgctgcgtcttattttcc) and PmBEAT37-RNAi-R1 (tgggcgcgccCCATGGtgagtgctgaaactacgagg) primers and the PmBEAT37-RNAi-F2 (gcaggtatttGGATCCtgagtgctgaaactacgagg) and PmBEAT37-RNAi-R2 (gactcacctaGGATCCgatgctgcgtcttattttcc) primers, respectively.

To study the subcellular localization of the PmBEAT proteins, the cDNA sequences of *PmBEAT34*, *PmBEAT36* and *PmBEAT37* were cloned into the pSuper1300-GFP vector using the In-Fusion method. The primers used were the same as for the pSuper1300 vector constructions.

### Enzyme extraction and assay

For comparison of the activity of benzyl alcohol acetyltransferase in the flowers of *P. persica* and the different varieties of *P. mume*, the crude protein samples were extracted using a protein extraction buffer containing 50 mM Tris-HCl (pH 7.5), 150 mM NaCl, 10 mM MgCl_2_, 0.1% NP-40 and a protease inhibitor cocktail (Roche). Then, 2 μg of crude protein was added to 1 mL of enzyme reaction buffer containing 50 mM Tris-HCl (pH 7.2), 3 mM 2-mercapto-ethanol, 0.5 mM acetyl-CoA and 1 mM benzyl alcohol. The assays were carried out in a 30 °C water bath for 1 h. The components were extracted with 0.5 mL ethyl acetate. Benzyl propionate was added as an internal standard, and the contents of benzaldehyde, benzyl alcohol and benzyl acetate were analyzed using GC-MS. The enzyme activity was calculated as the yield of benzyl acetate.

To detect the benzyl alcohol acetyltransferase activity of the PmBEATs, *PmBEATs* were transiently expressed in tobacco leaves by *Agrobacterium tumefaciens*-mediated transformation. The crude protein samples were extracted using a protein extraction buffer as described above. Then, 2  μg of crude protein was added to 1 mL of enzyme reaction buffer containing 50 mM Tris-HCl (pH 8.0), 0.5 mM acetyl-CoA, 1 mM benzyl alcohol, 0.01 mM MgCl_2_ and 1 mM DTNB. The assays were carried out in a 30 °C water bath, and the increase in absorbance at 412 nm was recorded using a spectrophotometer to detect the production of 2-nitro-5-thiobenzoic acid by the reaction of DTNB with free CoA^31^. The enzyme activity was calculated as the consumption of acetyl-CoA.

To verify the functions of *PmBEAT36* and *PmBEAT37* in floral synthesis, the purified plasmids were transformed into petal protoplasts of *P. mume*. After 30 h of incubation, the protoplasts were collected, and the endogenous components were extracted by vortexing in ethyl acetate for 15 min. Next, benzyl propionate was added as an internal standard, and the contents of benzaldehyde, benzyl alcohol and benzyl acetate were analyzed using GC-MS.

## Electronic supplementary material

Supplementary Data
