# Supplementary material for: Expansion of PmBEAT genes in the Prunus mume genome induces characteristic floral scent production
Source: Hortic Res. 2019 Feb 1;6:24. doi: 10.1038/s41438-018-0104-4 (PMC6355818; doi:10.1038/s41438-018-0104-4)
Supplement: Supplementary file 1 — Supplementary Data [file 41438_2018_104_MOESM1_ESM.docx]

**Fig. S1.** Amino acid sequence alignment of PpBEAT21 and PmBEAT38.

**
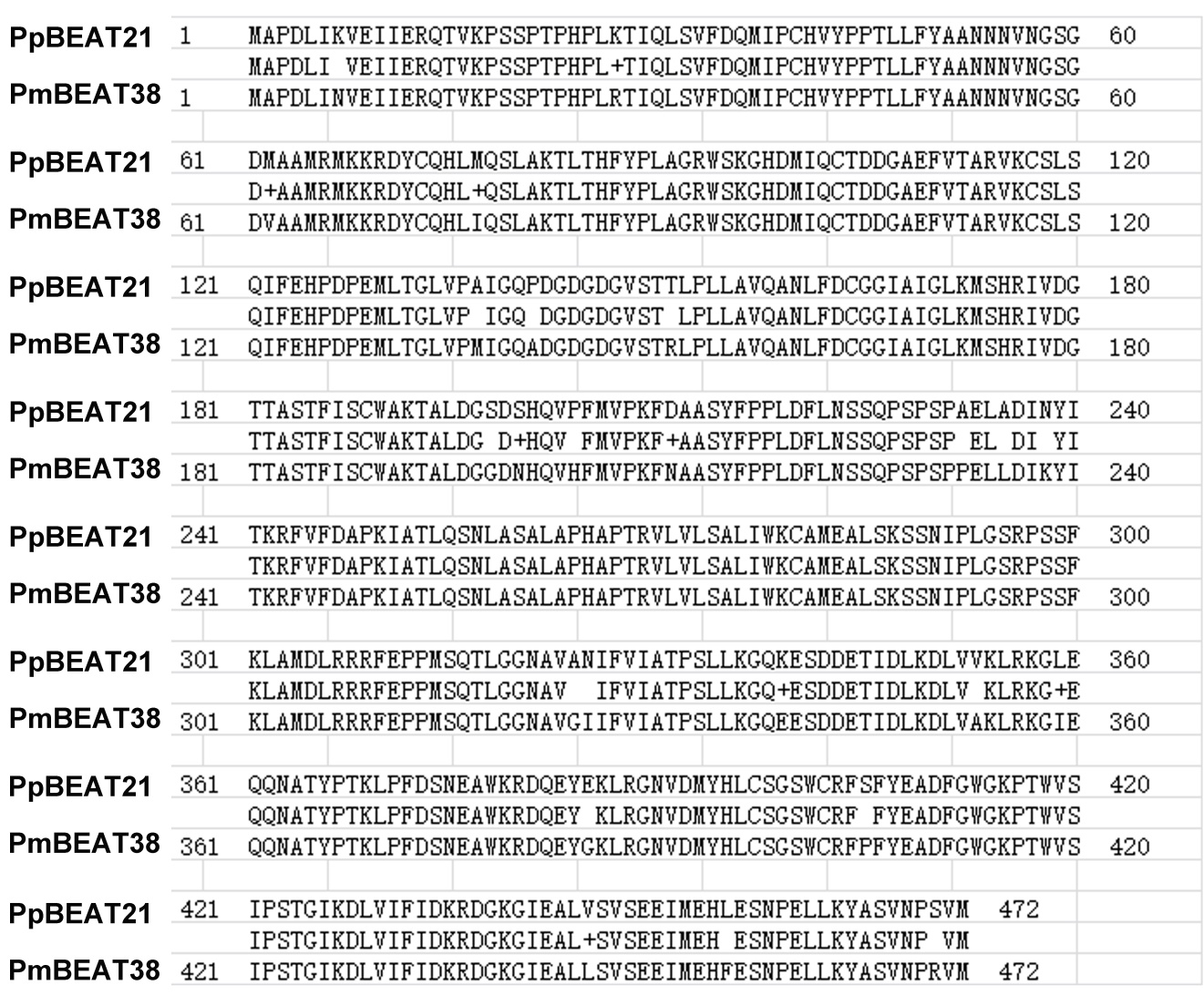
**

**Fig. S2.** The deletion in the coding sequence of *PmBEAT19*.


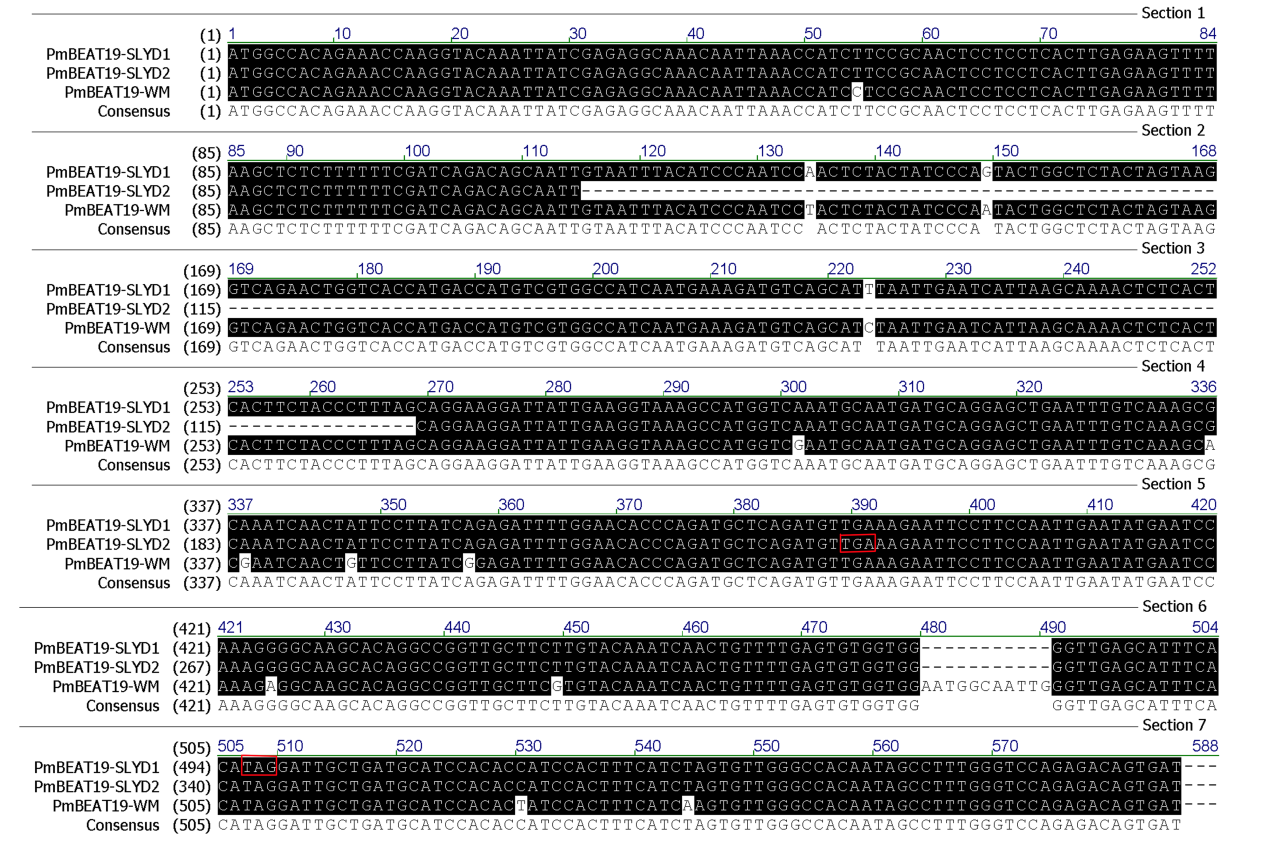


**Fig. S3.** The expression levels of *PmBEATs* and *PpBEATs* in different organs, as detected by semi-quantitative RT-PCR.


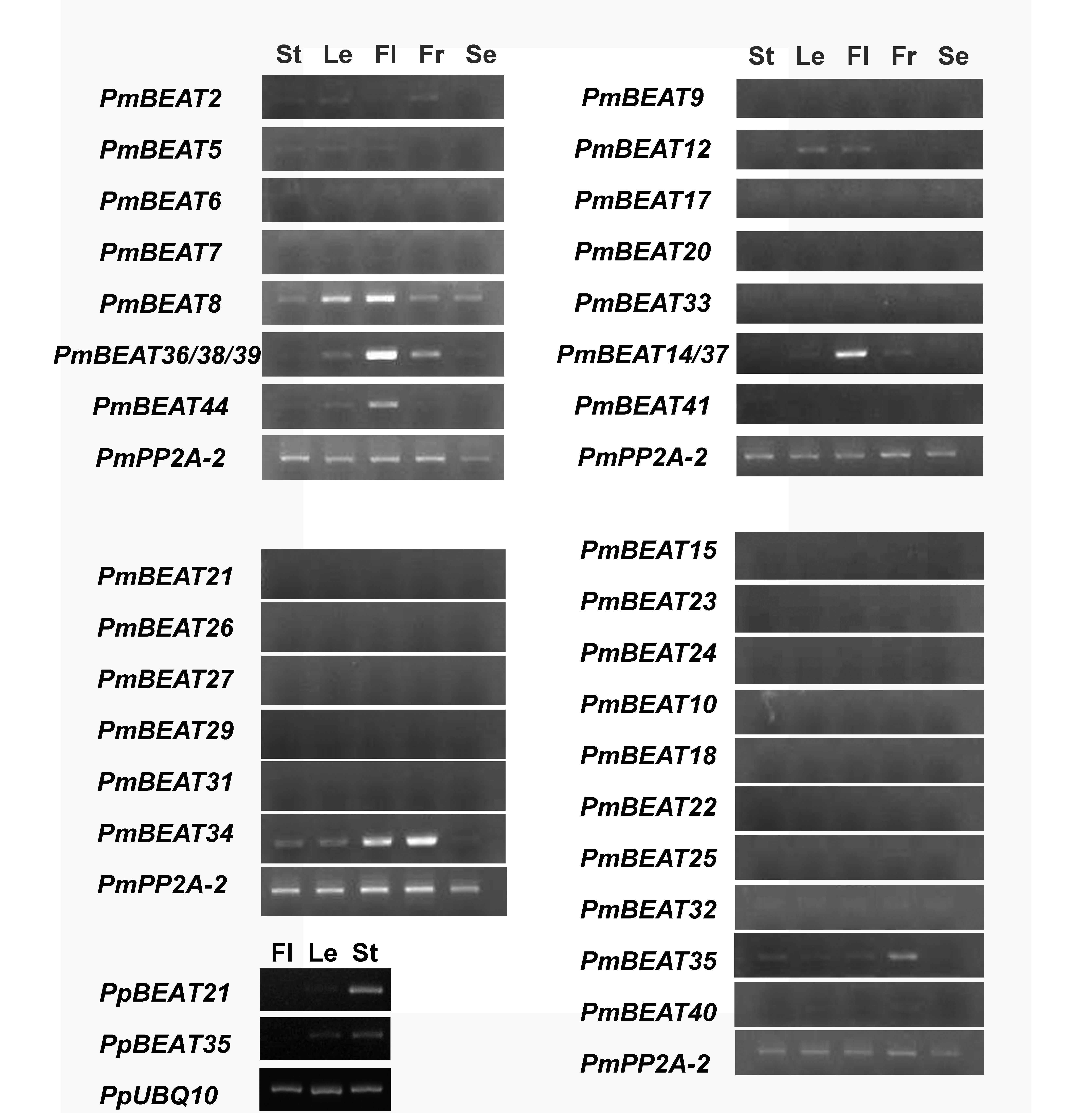


**Fig. S4.** The poly(A)-like tails of *PmBEAT* genes.

*PmBEAT11*

CACCAAAGAGGGTGGTGGAATAGAGGCTTATGTTAGTTTGGAGGAGGAAGTCATGGCTAAGTTTGAATGTGATGGCGAGTTGCTCTCCTATGTTGCTCCATCCGGTGGGGTGCTCCTAAGCTAAGGAAATAAATTTCTCTGTGTAACTATATGTTTGTGTTAGAAATTTCCTTATGTAACTGTAATATGTCTGAGTTATTTGCAGTTTCAGATTTCAATAATCGTCAAGCTTTTCTCAATTAAGGCCTACACTTTGAATATTAGTATATACAAAGTCTACAACAATATTTGGTTATAACTTGCTATTTTTCAAGTTGCAGGTTTCTTTGCCGTGTATGTATCTTAGATGACGACAAAATGTAAGTAGAGCCATAAATAGAGGAAAAAAAGAAGTCAATCGTCAAAACTAATTTCCTACCAACAAGTTTGCCTCTCGACCTAATGAACGTTGACTAAAATTTGTTTCCTCAGACTTTGATGGATAGGTTAACATCGTTTGACCAAAACCCCAACAGTTCAAGCATTTTTCAGAATGCAGTTGTTCAAGGGTGGAAGCCTCTGGTGTTTAAGGAACCTGTGCTTCCCAATGCTACTAATCTTTGCAGTATTGACGTGGTGGTTGCTTTCCAGGAAAAACTTCGTAGATTGTCTGGCCAGGTTATGTGCAATCTAAAGCTTCCATTCAGTGTTAATGGAACATCTTGGAATTCTTGCCAGACTAAAAAGTGCTCCGAAGTTTTTTATTATTGGGGTTTTAGTTTTAGGTTCTTGTAATTGAACTTTATAATTGAATTTTTAAATTCTTTCTTTGATAATATTTATTTAATATATGGATAAAATAGAATTTAATCAATAAAAGTACATACTATACAAATCGATTATATATGCAAGCACTCGCATGAAACTACCCATGCAAGTAGACATGGGGAAATGGGGGAAAATTGAAGGACAATTGTCTATTTAGAAATAAACGAATCCAAAATAGTGTGGAGAAGAAAAAATAATAGCCTATAATGATTAATTGCACCACATACTCTTCAAGTAACAAAAAAAAAAAAAATGAAAAGGAAAAAAGTGAGCTCAACAAAAATAAAAATTAAAGGAAAAATGAGGTTGCCTATGG

*PmBEAT14*

GAGTGAAGAAATCATGGAGCATTTTGAAAGCAATCCGGAGCTTCTTAAATATGCTTCTGTAAATCCAAGGGTCATGTAGCACTGTGTGTTAAGGTCGGAGTTTAGTCGAGTTTAATTAGTATTATTGTGTCGTGAGAATAATTATTTTCATTGCGCTAGCTGTGAGAATAATCACTTTCAATTGTTTTGTGAGAAGATGGTTATTGAATTTGTTGTCTTCATGAAAAGAAATATAATCAAATCAATATTATATTGCTCATATTATTATCCATCATTGGTTGTAAGAAACTAAAGATCTTACTGATAAAAAGAGAAAAAAAAAATCTACAATTGTTGTATAATAAACGAATGGTACTGGAATATAAAGGTTGCACATAAAGTAAATAAAACACAATATTTGACGAGGTTCGGCAAGTTGTG

*PmBEAT15*

CCGAAATCGAGCAAGTGTTATTAAGAAACCAACCCAAACCAAACTAATGAAGCTGGGGTCAGGTTCAGTTTGTTCGGTTTGCTTCTGAAGCTTATGAAAAAATAGTTGGCATTAAAATCTATAAGGCTTGCTATAAAATAAGAAAAGCCCTTAGAGCTAAAAGCCACTTTAATTTTTTTATTTTTCTTTGTGTGTATTGTTTTGCAATTTAAGTTGCTTCTTGACGCTTTTGCATCCTTGTAATTGTTTTATCAATAAATTTCTCAACCAAAAAAAAAAAATAATAATAAAAGAAAAAAAAAAAGAAAGTTAACTGACTAGTGACTATTGTAGAAAGGTGAACAATGTGATTTTGGAGTGAATGAATAGTGTACGGTTAAAAAAATTGTTGGTCTTTGTCAGATCTGAGCCGTTCA

*PmBEAT24*

ACGGCATGGGCATAGAAGCGTCATTGACTTTGAAGAAAGACGACATGGCCATGATTGAAAGTAGTAAGGAGTTGCTTGCATATGCAACCCTGAACCCGACTGTGATTTAGTTAGTCTTTTCTTACTTGAATATCTCAACGGTCTATGTTTAATCGTATATGATTAATAACAAATCATTGTTAATCATACACGTGTATTAAACATGAATAATACGTATGTATGTTTACAATATGAGTATTGTACATTTGCTTTTTAAAAAACATGTATTTTACTGAGTCTTTAAGACATGTACAGAAAACGGATGTACTATTGAAAAATACATATGTACATGTATAATATGAGTATTAAACATAAAAAATGCTAAATTCTTTATACGCATAATAACTCTAGGAAAGTAAAAAAATCAAAAGGGGACAATAGAAACTCCGGTAATGGCCTAATAGTGATGAATAATGATCTAAACTACTCTTATCGATAAAAGAAAATCAGGAAAAAAATAAAAAATAATAAAAAAAGGATCCTAGTATATATATAAAGAATTGGCTATATTGTTTATTAAAAAGAAAACCTGGTAACACCACGTCTATACTTTTTTTAAAAAATAAATAAATAAAAGAAATACCTGAGTATAGC

*PmBEAT26*

GGCGTAGGCATAGAAGTCTACTTGGCTTTCAAAGAAGAAGACATGGCCCTAATTGAAAGCAATGAGGAGCTGCTTGCCTATGCTTCTCTGGTTTAATTAGTCTCTCCTCTTGTGCTTCTTAGATGAATGCTAAGGCGGATCTCTGACGATAATCACTTATAATAGTTTGTATGCTTGTATTGTTTGTGCTTTCATTGCATTTTTCCCCTTTCAATTATGTACTTCAAAAAAATATGAATGTTGAGATTTGTTTCACCCCCTTGAGCGTGAATTAATCATAACGTACCGAACTCGTCAACCAAATCCTAAAAACCTCATTTGTAACTAATAAATTATTATTAGCTAGGTACGCTTTAATTCTGGTTGGCATTCCATTTTGATTGAATTGCAAGAGGTTAGCCAGATCCAATCACATACTTCTTTAATGCTCTAACCACATACAATAAGCTTTGAGAAAAATAATACAATAGTACTAGATATGGACAATACAATAAATTAATGAAGTTGACTTCCAGAAATCAACATCTGGGTCTTTTTATAATAGGGAGGGTCTTTTTTATGAAACTCAAGTTTGGACTGTTGAGGAAGAGATGGTTTCAGAAATCAAGGTTGAAGTGATTCACAAAAAAAACAAAAACAAAAAAAACAATCCAACCATCCTCCCCTGCTCCTCACCACCTTAGAAATGCCAACATCTATGTTTTTTCGCTAGTAAAAACAGTGGCAACAGTCACCATTACTTGGTGACATTTGTGGCCTTGAAG

*PmBEAT27*

AAGCGTCCTTGACTTTGAATGAAGAAGTCATGGCTATATTTGAAAGCAATAAGGAACTGCTTGCATATGCTTCTCTGAATCCGTCTGTCATTTAATTAGTACCAAAAAGGAAAATTAAATAACAAAAGGCCTCCTCCTTGTGATTCTTAGATGAACATTATTGCTGGAGCCTTGATGATCTTTTCATTGTTTTCGTATTTGTTCACTTAAGTTTGTACGAAAAATAAAAGAGATTTTCTATTTAAACCCCACACTTCTCTTAGTTCACCCCATGTTCTAAGTTCACCCCAACTTTTAATTTAAATTTTCACAATTTCTAAGAAAACCCCACTATCTTCCCAAACTACCCTTAAATACACCCCAAACTAAAAAAATAAAAACTAAAAGAGATTTTCTATTTAAATCCCACAATTTTCTTTGTTCTCAGCAAAAAAAAAAAAAACACAAACAAATGAAACCCCAAATCTGAAATTTAATATTTTCTAGAGAAAGAGAACAACTTTACCTCTCTCTAAGAACTATAACTGGAATTCTAACACTTGGGTATTGTTGAGTT

*PmBEAT36*

ATGCGTTTTATCTGATTGATAAAAGAGATGGCAAGGGAATAGAAGCATTGTTGAGTTTGAGTGAAGAAATCATGGAGCATTTTGAAAGCAATCCGGAGCTTCTTAAATATGCTTCTGTAAATCCAAGGGTCATGTAGCACTATGTGTTAAGGTCGGAGTTTGGTCGGAGTTTAATTAGTATTGTTGTGTTGTAAGAATAATTATTTTCATTGCGCTAGCTGTGAGAGTAATCACTTTCGATTGTTTTGTGAGAAGATGGTTATTGAATTTGTTGTCTTCATGAAAAGAAATATAATCAAATAAATATTATATTGCTCATATTATTATGCCTCATTGGTTGTGAGAAAACTAAGGTCCGTTTAGTTCATGGAATGAATTTGAGGGGAAATCATTTTCCATGTCATTCCCCAAGGAATGGGA

*PmBEAT37*

TTTTGATTGATACAAGAGATGGCAAGGGAATAGAAGCAATGTTGAGTGTGAGTGAAGAAATCATGGAGCATTTTGAAAGCAACCCGGAGCTGCTTAAATATGCTTCTGTAAATCCAAGGGTCATGTAGCACTAAGTGCTAATTAAGGAAGGTCTGAGTTTAGTCTAATTGCTATTGTTGTGATTCTGTTGTCAGAATAATCATTTTCAATTCTATTGTGAGAATAATATTCCAAAATCTAAGTATTAAAGTCGAGTTTAGTGTAATTAGTATTGTCATAATACGTGAGAATAATCATTTTCACTCTGATGTGCAATCTTTGGAAAATACTCTTTTTTTTCGTGGCCAAGGATTAGTCTTTATAGTATACGTCTAGCTGTTATCCTAGCTATTTGTTCTAAAGTTGGGGACATCAAAGGTTGATCCTAACCGTCAGTCGGAGCCACTTTACAAATAATCTCTCACTGATTTATTGGCGCTGCGCTTTTTTTCTTGGCCGGGACGAGTTATAATAGAATACGTCTTGTTTATCTCGACGAAATGGGCGGAATGGCTATCGCAATTCCAAAAATATTCACGAAACTTAGAGAGCACACTACCAAAAAATAAAAATAAAAAAAGTGGCCACAGTCACCATTATTTGGTGCAATGGCGGATCTAGGATTTTTTACAGGGGTGGGCCAAATTTTTTTTTGGAA

*PmBEAT39*

AAGAAATCATGGAGCATTTTGAAAGCAACCCGGAGCTGCTTAAATATGCTTCTGTAAATCCAAGGGTCATGTAGCACTATGTGGAAAAAGTAATAAATTTGAATTATGTTGTGAGAATAATCACTTTCAATTCTGTTTGTGAATAATCTTATGGTCCGTTTGGATGAAGTATTCCAAAATCTAAGTATTAGTTTAGTTTAGTTAGTATTGTTGTAATGTCAGAATAATCATTTTCATTGTGAAGTGAAAAAAAATAAAAAAATAAAAATTATCTCCTCCTCAACCTTTGCCTTCCCCAAGACCGAAACCCTATCTCCAGAAACCCAAGATGAAACTCCTTTATTCTATT

*PmBEAT41*

CAAAGAGGGTGGTGGAATAGAGGCTTATGTTAGTTTGGAGGAGGAAGTCATGGCTAAGTTTGAATGTGATAGCGAGTTGCTCTCCTACGTTGCTCCAACCGGTCGGGTGCTCCTAAGCTAAGGAAATAAATTTCTCTGTGTAACTATATGTTTGTGTTACAAATTTCCCTATGCAGTTTCAGGTTTCAATAATCGTTATGATATTATATCAGAATCGTTTACAGTGACGATTCATCAACCACAATTGAGGTCTTCTCTCCATAATAAAATTGAGGTCTTCTTCCCATAATTAAATTAAAATTTTAAAAACTCTTATTACAAGATAAGGTTTGATATGGCTTTGATGACCGGATAGGCTGAGAAGGTGCTCAAGAAGAAGAAAAATGTGGAGAAGATAATGGTGATCATGCATCGTTGATTGGTCTGTAATTAGGACTGAAGTCTAAATTTTTTTTGTTAAATAAAGGGAGCTGATTTCCCCACTCCCATTTTCTCCACTTATACTCCGTTTTATTTTTTAATACTTTTTAATTAATTTTGTTCTTTTTTGTTCTCTCTTTCCTATGCTACCCTTACTCTACATTATTTTCTTTTTACTTCACTTTTATATTATTTCTTCTTTTTTCCCCATCAATTTTCAAAATTAATCAAAATAAAAAAGAGAAAACAAAATATATAAAAATTAATCAAAATAAAAAAGAGAAAACAAAATATATAAAAAAACCCAGCCCCTCCTGTGTTGTACCCAACCTGTGCCCTCCCTCGCCACCCTGCCCAGCCACCACCTGCTCTCTTCCTCCATTTATCTTTCTTTCTTTTTCCATACCCACCAATCTCTCTGTC

*PmBEAT42*

CCATATTTGAGTCTGACGAAGAGCTCCTTGCATATTTGGTTCCCTCAACCCCAGCACAAACAATGTTTGATATCAAGTCTTCAACAAAGTTGAAATCTAATATGTGAAAAAGGTGTGAATTATCTCTTCCCGTGGATTATAAATAAATAAACAAATAAGTTTTGTGTCACGCATGAAGAAAAATACTACCAGACTAGTAGCTAATTTGTAATTAAGTGTCATATAAAATATTAGAGAGTGTATTGAGTTTCAATCTTTTGGAACGGATTGCTTTTTTATT

*PmBEAT44*

TGGAAGAAGAACACATGGCCATATTTGAACGCAATGAGTACCTGCTTGCATATGCTTCTATGAATCCGAGTGTCGTTTAATTAGTACCCTCTTATGCTTCTTAGAGGGATATGCTGTTGTGGATCATTGATGATATCTCTCATAATCGTTTAGATGTTTGCACCATATTTGTGCTTCATCGCATTTTCCCTTATTGTTTCGTTGTTTATCATATGTTGTTTTCAATTCAATAATGCTATTTAGACACATAAGACTGACCACATTAGCTGGCCACCTTTTGTGGCAGCTGACGTGGTCATGCCGTGTCAATTATAGCAGCTCAATATGATTATTCTTGAAGTAACGAGATCGTCTCCAACTTAGTCTGTTCGCTGGTTTATATGTGCTTAGCTTTTTGACGAGTTCCTTGCATGTGTGCAACGTCACTTTTCTCTTCTACTCCATGTTCAAGAATCAGGCCTTTAAATCTTCGAATTTTTTTTCGTAATTCATATGAAACTTAAGTTCATTAAGGTAACATGTGTAATTTTTATTACAATTATTTCAAATAAAGAAAAGAAAATTTAAAAATTTAAAAAAAAAATAAAAAAAAAATAATATTTCGAGAGGTTTGCACGACGCAAAGAGATCATCCACGTCGCGCAAGTTGTCCGGATATACTAGAAAAAATAATATTAAATTTATGTTTCAAAACATAGGCGA

**Table S1.** The sequences of primers used in gene amplification.

| Gene | Primers (5’-3’) | |
| --- | --- | --- |
| PmBEAT2 | Amp-F: atgatgaggatccaagttat | Amp-R: ctaaatcttagcatcttggg |
| PmBEAT4 | Amp-F: atgaaggttgaagttgaagt | Amp-R: ttagcttaggagcacccgac |
| PmBEAT5 | Amp-F: atgaaggttgaggttgaagt | Amp-R: tcagcaaccacttagagaaa |
| PmBEAT6 | Amp-F: atgaaggttgaagttgaagt | Amp-R: tcacttgagcccatgtggag |
| PmBEAT7 | Amp-F: atgaagattgaagttgaagt | Amp-R: ttaaagacgtgcaagtggat |
| PmBEAT8 | Amp-F: atgaagattgaagttgaagt | Amp-R: tactttaagtgtggtgcaa |
| PmBEAT9 | Amp-F: atggcccaggagatgaaaat | Amp-R: ctaaagagaagatctccttg |
| PmBEAT10 | Amp-F: atggcccaggagatgaaaat | Amp-R: ctaaagagaagatctccttg |
| PmBEAT11 | Amp-F: atgaaggttgaagttgaagt | Amp-R: ttagcttaggagcaccccac |
| PmBEAT12 | Amp-F: atggaagttgaaattatatc | Amp-R: tcaaatgacactcggattca |
| PmBEAT14 | Amp-F: atggccacagacttggtcaa | Amp-R: ctacatgacccttggattta |
| PmBEAT15 | Amp-F: atgaacgttcaagtggaagt | Amp-R: tcagaagcaaaccgaacaaa |
| PmBEAT16 | Amp-F: atggccacagaaatcaaggt | Amp-R: tcagttggtgaccctagggt |
| PmBEAT17 | Amp-F: atgaatgttcgagttgaagt | Amp-R: ttacaaacggctgattttgg |
| PmBEAT18 | Amp-F: atggagtttgaaataatcaa | Amp-R: ttacagagcagacttccttg |
| PmBEAT19 | Amp-F: atggccacagaaaccaaggt | Amp-R: ttaagtggtgatcaaacttg |
| PmBEAT20 | Amp-F: atgacttcagaaatcaaggt | Amp-R: ttacacgacactcggattca |
| PmBEAT21 | Amp-F: atggttacagagatgaaggt | Amp-R: ttacaggtcgagactcggat |
| PmBEAT22 | Amp-F: atgacttcagagatcaaggt | Amp-R: ttacacgacacacggattca |
| PmBEAT23 | Amp-F: atggcttcagagatcaaggt | Amp-R: tcaaagcttaggtatgagtt |
| PmBEAT24 | Amp-F: atggcttcagagatcaaggt | Amp-R: ctaaatcacagtcgggttca |
| PmBEAT25 | Amp-F: atggtttcagagatcaaggt | Amp-R: ttaaatgacagttggattta |
| PmBEAT26 | Amp-F: atggcttcagagatcaaggt | Amp-R: ttaaaccagagaagcatagg |
| PmBEAT27 | Amp-F: atgggtttagggattagggt | Amp-R: ttaaatgacagacggattca |
| PmBEAT29 | Amp-F: atggtttcagagatgaaggt | Amp-R: ttaataaatgacagtcggat |
| PmBEAT31 | Amp-F: atggctccagaggtaaaggt | Amp-R: ctaaattacacttggattca |
| PmBEAT32 | Amp-F: atggcctcacaaatcaaggt | Amp-R: tcagtaggtgaccctaggat |
| PmBEAT33 | Amp-F: atggccacagaaatcaaggt | Amp-R: tcagttggtgaccccagggt |
| PmBEAT34 | Amp-F: atggcttcagaaatcaaagt | Amp-R: ttaaacgacactcggattca |
| PmBEAT35 | Amp-F: atggcttcagaaatcaaagt | Amp-R: ttaaacgacactcggattca |
| PmBEAT36 | Amp-F: atggccacagacttgatcaa | Amp-R: tacatgacccttggattta |
| PmBEAT37 | Amp-F: atggccacagacttgatcaa | Amp-R: ctacatgacccttggattta |
| PmBEAT38 | Amp-F: atggccccagacttgatcaa | Amp-R: ctacatgacccttggattta |
| PmBEAT39 | Amp-F: atggccacagacttgatcaa | Amp-R: ctacatgacccttggattta |
| PmBEAT40 | Amp-F: atggccacagacttgatcaa | Amp-R: ctacatgacccttggattta |
| PmBEAT41 | Amp-F: atgaaggttgaagttgaagt | Amp-R: ttagcttaggagcacccgac |
| PmBEAT44 | Amp-F: atgggttcagaagtcaaagt | Amp-R: ttaaacgacactcggattca |

**Table S2.** The sequences of primers used in semi-quantitative RT-PCR.

| Gene | Primers (5’-3’) | |
| --- | --- | --- |
| PmPP2A-2 | Sem-F: atgccggctcacgcggatctg | Sem-R: ttacaaaaaataatctggagtc |
| PmBEAT2 | Sem-F: ttcctaaacagctgggcttc | Sem-R: ccaccaaattcttaaacggg |
| PmBEAT5 | Sem-F: gaggacagttcaggttttaac | Sem-R: ccaccaggttcttgaatgtc |
| PmBEAT6 | Sem-F: caacaagatgaacatatagtgtg | Sem-R: gttattgaaagtgaggggag |
| PmBEAT7 | Sem-F: caaaagttctatagtgggtcc | Sem-R: tccatgaggcttctcacttg |
| PmBAET8 | Sem-F: tcaaggtctcaaaaacccag | Sem-R: cacgatgatgctattctcattc |
| PmBEAT9 | Sem-F: ctcatttggaagtgtgcaac | Sem-R: aacattcttgactacaccacc |
| PmBEAT10 | Sem-F: cctcatttggaattgtgcaatg | Sem-R: caaacaagcaacattcttgatg |
| PmBEAT12 | Sem-F: gccagaagccatgattgag | Sem-R: catggcacagctagcaatag |
| PmBEAT14/37 | Sem-F: ctcatcacagccatcatcag | Sem-R: ccaatactaggaacgcttac |
| PmBEAT15 | Sem-F: ggatcaccagctataattgg | Sem-R: gtcgaggaaaactattatgtcc |
| PmBEAT17 | Sem-F: aggagaccggtatcacaaag | Sem-R: gcaataaccgacttcactag |
| PmBEAT18 | Sem-F: tcattccagcagtcttcagtg | Sem-R: ttgtttgaagcagcagggac |
| PmBEAT20 | Sem-F: ttggctcagccaatactgac | Sem-R: actagggttagtcatccatg |
| PmBEAT21 | Sem-F: ataatggttctgcccttgac | Sem-R: ggattcagagaagcgtatgc |
| PmBEAT22 | Sem-F: atattgccgcagttgccag | Sem-R: tcctcgttgctttcaatcac |
| PmBEAT23 | Sem-F: ccactgctactactgatgtac | Sem-R: gtttgtgacgaacctcatag |
| PmBEAT24 | Sem-F: gccatcagtgtgttgtcaag | Sem-R: catatccatcaatgtgaccatg |
| PmBEAT25 | Sem-F: aagtccaaagctgccacc | Sem-R: catcgctcaaatccatcaac |
| PmBEAT26 | Sem-F: acgccaaccaactgtgg | Sem-R: cttctttgaaagccaagtagac |
| PmBEAT27 | Sem-F: aattgcttccagctgatatgg | Sem-R: agccaacctttgaacatcttc |
| PmBEAT29 | Sem-F: tcacctcaacctgcgttg | Sem-R: ttcttcagtcctgtactctg |
| PmBEAT31 | Sem-F: cagcgcaaactctggtatc | Sem-R: cttggaatctacactagagg |
| PmBEAT32 | Sem-F: agaggatgccgccaaag | Sem-R: gattgttagcttcaacaggaac |
| PmBEAT33 | Sem-F: catccgtggaagtcgataag | Sem-R: gttacattgttaattgcaacagc |
| PmBEAT34 | Sem-F: catccacgtggaatcaagc | Sem-R: ttcactcctagagaaagtcac |
| PmBEAT35 | Sem-F: gactgtggaaagtgatgaag | Sem-R: tgtgacagagactcatccac |
| PmBEAT36/38/39 | Sem-F: gagggcatcaagtacataac | Sem-R: ccattgccatgccttaatag |
| PmBEAT40 | Sem-F: tcatcacagccatcaccatc | Sem-R: ccttacaaccagtactagga |
| PmBEAT41 | Sem-F: ttgatggtagcgttggcatc | Sem-R: aaggttcttgtaggtgagtg |
| PmBEAT44 | Sem-F: ccgatcgtggagttgattag | Sem-R: tcttggattcattgcgacag |
| PpBEAT21 | Sem-F: aaactctcactcacttctacc | Sem-R: ccttctacgcaagtccatag |
| PpBEAT35 | Sem-F: tacatacaacaacgctgcac | Sem-R: ggacatcatcagatctgtcg |
| PpUBQ10 | Sem-F: tctctctcaagatgcagatc | Sem-R: gtatccgagctttcaacctc |

**Table S3.** The sequences of primers used in real-time PCR.

| Gene | Primers (5’-3’) | |
| --- | --- | --- |
| PmBEAT8 | RT-F:tgactgctatgcgtatca | RT-R: ttccttgacattaccaactg |
| PmBEAT14 | RT-F: ctcattcctcgagaaccac | RT-R: gtgagagttttagctaatgacc |
| PmBEAT34 | RT-F: gtctacggaaagtgaagtg | RT-R: caatgataggtctcagtgttat |
| PmBEAT35 | RT-F: aggtcaactgtcccatatc | RT-R: cctgcctctgattgtttg |
| PmBEAT36 | RT-F: cgagtatggaggaatagcaa | RT-R: tcatcgtcaccaacagaa |
| PmBEAT37 | RT-F: tgacggtgttgccac | RT-R: atcatcgtgacaatcatcaag |
| PmBEAT38 | RT-F: ctggagggcatcaagtaca | RT-R: cgcaagtccatagccattt |

**Table S4.** Information on *PmBEAT* and *PpBEAT* genes.

| Name | Gene ID | Name | Gene ID | Name | Gene ID |
| --- | --- | --- | --- | --- | --- |
| PmBEAT1 | Pm006849 | PmBEAT39 | Pm011503 | PpBEAT3 | ppa005255m |
| PmBEAT2 | Pm006850 | PmBEAT40 | Pm016771 | PpBEAT4 | ppa005318m |
| PmBEAT3 | Pm006854 | PmBEAT41 | Pm025400 | PpBEAT5 | ppa005418m |
| PmBEAT4 | Pm006857 | PmBEAT42 | Pm025606 | PpBEAT6 | ppa005452m |
| PmBEAT5 | Pm006858 | PmBEAT43 | Pm025612 | PpBEAT7 | ppa005455m |
| PmBEAT6 | Pm006859 | PmBEAT44 | Pm026103 | PpBEAT8 | ppa005502m |
| PmBEAT7 | Pm006860 | PmBEAT45 | Pm002535 | PpBEAT9 | ppa005563m |
| PmBEAT8 | Pm006862 | PmBEAT46 | Pm003732 | PpBEAT10 | ppa005630m |
| PmBEAT9 | Pm006864 | PmBEAT47 | Pm008312 | PpBEAT11 | ppa005635m |
| PmBEAT10 | Pm006868 | PmBEAT48 | Pm008809 | PpBEAT12 | ppa005640m |
| PmBEAT11 | Pm006869 | PmBEAT49 | Pm008812 | PpBEAT13 | ppa005715m |
| PmBEAT12 | Pm006894 | PmBEAT50 | Pm013820 | PpBEAT14 | ppa005774m |
| PmBEAT13 | Pm010966 | PmBEAT51 | Pm013822 | PpBEAT15 | ppa005872m |
| PmBEAT14 | Pm010967 | PmBEAT52 | Pm013827 | PpBEAT16 | ppa005873m |
| PmBEAT15 | Pm010968 | PmBEAT53 | Pm013834 | PpBEAT17 | ppa005910m |
| PmBEAT16 | Pm010971 | PmBEAT54 | Pm014352 | PpBEAT18 | ppa005943m |
| PmBEAT17 | Pm010973 | PmBEAT55 | Pm016033 | PpBEAT19 | ppa005955m |
| PmBEAT18 | Pm010974 | PmBEAT56 | Pm016036 | PpBEAT20 | ppa014960m |
| PmBEAT19 | Pm010975 | PmBEAT57 | Pm016042 | PpBEAT21 | ppa016640m |
| PmBEAT20 | Pm010976 | PmBEAT58 | Pm016045 | PpBEAT22 | ppa017691m |
| PmBEAT21 | Pm010977 | PmBEAT59 | Pm016046 | PpBEAT23 | ppa019948m |
| PmBEAT22 | Pm010979 | PmBEAT60 | Pm016048 | PpBEAT24 | ppa021452m |
| PmBEAT23 | Pm010980 | PmBEAT61 | Pm016126 | PpBEAT25 | ppa021495m |
| PmBEAT24 | Pm010984 | PmBEAT62 | Pm016482 | PpBEAT26 | ppa023329m |
| PmBEAT25 | Pm010985 | PmBEAT63 | Pm016484 | PpBEAT27 | ppa023555m |
| PmBEAT26 | Pm010986 | PmBEAT64 | Pm018886 | PpBEAT28 | ppa023735m |
| PmBEAT27 | Pm010988 | PmBEAT65 | Pm018954 | PpBEAT29 | ppa024073m |
| PmBEAT28 | Pm010989 | PmBEAT66 | Pm020331 | PpBEAT30 | ppa025470m |
| PmBEAT29 | Pm010990 | PmBEAT67 | Pm023001 | PpBEAT31 | ppa025565m |
| PmBEAT30 | Pm010992 | PmBEAT68 | Pm023027 | PpBEAT32 | ppa025918m |
| PmBEAT31 | Pm010994 | PmBEAT69 | Pm026398 | PpBEAT33 | ppa025976m |
| PmBEAT32 | Pm010996 | PmBEAT70 | Pm026402 | PpBEAT34 | ppa026050m |
| PmBEAT33 | Pm010998 | PmBEAT71 | Pm026403 | PpBEAT35 | ppa026262m |
| PmBEAT34 | Pm011001 | PmBEAT72 | Pm026406 | PpBEAT36 | ppa026934m |
| PmBEAT35 | Pm011005 | PmBEAT73 | Pm027450 | PpBEAT37 | ppa026936m |
| PmBEAT36 | Pm011009 | PmBEAT74 | Pm030674 | PpBEAT38 | ppa1027182m |
| PmBEAT37 | Pm011010 | PpBEAT1 | ppa004875m |  |  |
| PmBEAT38 | Pm011011 | PpBEAT2 | ppa004937m |  |  |
